# Prospective high-throughput genome profiling of advanced cancers: results of the PERMED-01 clinical trial

**DOI:** 10.1186/s13073-021-00897-9

**Published:** 2021-05-18

**Authors:** François Bertucci, Anthony Gonçalves, Arnaud Guille, José Adelaïde, Séverine Garnier, Nadine Carbuccia, Emilien Billon, Pascal Finetti, Patrick Sfumato, Audrey Monneur, Christophe Pécheux, Martin Khran, Serge Brunelle, Lenaïg Mescam, Jeanne Thomassin-Piana, Flora Poizat, Emmanuelle Charafe-Jauffret, Olivier Turrini, Eric Lambaudie, Magali Provansal, Jean-Marc Extra, Anne Madroszyk, Marine Gilabert, Renaud Sabatier, Cécile Vicier, Emilie Mamessier, Christian Chabannon, Jihane Pakradouni, Patrice Viens, Fabrice André, Gwenaelle Gravis, Cornel Popovici, Daniel Birnbaum, Max Chaffanet

**Affiliations:** 1 Laboratory of Predictive Oncology, Department of Medical Oncology, Centre de Recherche en Cancérologie de Marseille (CRCM), Institut Paoli-Calmettes, INSERM UMR1068, CNRS UMR725, Aix-Marseille University, 232 Boulevard Sainte-Marguerite, 13009 Marseille, France; 2 Department of Medical Oncology, Institut Paoli-Calmettes, Marseille, France; 3 Biostatistics Unit, Institut Paoli-Calmettes, Marseille, France; 4 Department of Medical genetics, Hôpital Timone Enfants, AP-HM, Marseille, France; 5 Aix-Marseille University, Inserm, U1251-MMG, Marseille Medical Genetics, Marseille, France; 6 Department of Imaging, Institut Paoli-Calmettes, Marseille, France; 7 Department of Biopathology, Institut Paoli-Calmettes, Marseille, France; 8 Department of Surgical Oncology, Institut Paoli-Calmettes, Marseille, France; 9 Biobank, Department of Hematology, Institut Paoli-Calmettes, Marseille, France; 10 Department of Clinical Research and Innovation, Institut Paoli-Calmettes, Marseille, France; 11 Department of Medical Oncology, Gustave Roussy Cancer Campus, UMR981 Inserm, Villejuif, France; 12 Paris Sud University, Orsay, France; 13 Department of Oncogenetics, Institut Paoli-Calmettes, Marseille, France

**Keywords:** aCGH, Advanced cancers, Mutation, PERMED-01 trial, Precision medicine, Sequencing, t-NGS, WES

## Abstract

**Background:**

The benefit of precision medicine based on relatively limited gene sets and often-archived samples remains unproven. PERMED-01 (NCT02342158) was a prospective monocentric clinical trial assessing, in adults with advanced solid cancer, the feasibility and impact of extensive molecular profiling applied to newly biopsied tumor sample and based on targeted NGS (t-NGS) of the largest gene panel to date and whole-genome array-comparative genomic hybridization (aCGH) with assessment of single-gene alterations and clinically relevant genomic scores.

**Methods:**

Eligible patients with refractory cancer had one tumor lesion accessible to biopsy. Extracted tumor DNA was profiled by t-NGS and aCGH. We assessed alterations of 802 “candidate cancer” genes and global genomic scores, such as homologous recombination deficiency (HRD) score and tumor mutational burden. The primary endpoint was the number of patients with actionable genetic alterations (AGAs). Secondary endpoints herein reported included a description of patients with AGA who received a “matched therapy” and their clinical outcome, and a comparison of AGA identification with t-NGS and aCGH *versus* whole-exome sequencing (WES).

**Results:**

Between November 2014 and September 2019, we enrolled 550 patients heavily pretreated. An exploitable complete molecular profile was obtained in 441/550 patients (80%). At least one AGA, defined in real time by our molecular tumor board, was found in 393/550 patients (71%, two-sided 90%CI 68–75%). Only 94/550 patients (17%, 95%CI 14–21) received an “AGA-matched therapy” on progression. The most frequent AGAs leading to “matched therapy” included *PIK3CA* mutations, *KRAS* mutations/amplifications, *PTEN* deletions/mutations, *ERBB2* amplifications/mutations, and *BRCA1/2* mutations. Such “matched therapy” improved by at least 1.3-fold the progression-free survival on matched therapy (PFS2) compared to PFS on prior therapy (PFS1) in 36% of cases, representing 6% of the enrolled patients. Within patients with AGA treated on progression, the use of “matched therapy” was the sole variable associated with an improved PFS2/PFS1 ratio. Objective responses were observed in 19% of patients treated with “matched therapy,” and 6-month overall survival (OS) was 62% (95%CI 52–73). In a subset of 112 metastatic breast cancers, WES did not provide benefit in term of AGA identification when compared with t-NGS/aCGH.

**Conclusions:**

Extensive molecular profiling of a newly biopsied tumor sample identified AGA in most of cases, leading to delivery of a “matched therapy” in 17% of screened patients, of which 36% derived clinical benefit. WES did not seem to improve these results.

**Trial registration:**

ID-RCB identifier: 2014-A00966-41; ClinicalTrials.gov identifier: NCT02342158.

**Supplementary Information:**

The online version contains supplementary material available at 10.1186/s13073-021-00897-9.

## Background

During the last decades, the development of molecularly targeted therapies (MTT) directed against oncogenic drivers led to major progresses in the treatment of advanced cancers. Examples include inhibitors of EGFR and ALK in lung cancer, or ERBB2 and PIK3CA in breast cancer. In most of FDA-approved MTTs, which are used in clinical routine for solid cancers and target ~ 50 oncogenic drivers, the alteration of the target protein is diagnosed at the DNA level (amplification, mutation, translocation,…) and represents a relatively frequent event for the corresponding cancer. High-throughput molecular profiling, notably next-generation sequencing (NGS), improved our knowledge of oncogenesis and revealed the complexity of the genomic landscape of primary tumors. Today, more than 400 oncogenic drivers exist across cancers [1], but only a small fraction of them are targeted by the currently approved therapies; major research efforts are ongoing to develop MTT directed against the yet untargeted drivers. Most cancer types, including the most frequent, display a few drivers with relatively high frequency, but also many drivers with very rare occurrence and shared with other cancer types. These potentially actionable very rare alterations provide opportunities for therapeutic targeting across different cancer types. Even if the functional value of an alteration depends on the cancer type [2], impressive tumor responses to therapies directed against a rare alteration have been reported in nearly all cancers [2–5].

Such observations were the basis for the development of precision medicine in oncology, in which the therapy is delivered according to the molecular alteration identified in the patient’s tumor [6]. Since one decade, the development of MTT and the molecular segmentation of cancers coincided with technological advances in high-throughput molecular profiling that became consistent with real-time clinical use, further boosting the concept of precision medicine [7]. The first prospective trials designed to assess the value of molecular profiling for tailoring therapy in a pathology-independent way showed its feasibility in patients with advanced cancer [8–13]. They used conventional molecular techniques and/or limited gene sets and/or archival samples. Because of the logistics complexity, only some expert centers have set up NGS-based screening trials [14–23]. Today, the clinical benefit of precision medicine remains unproven [24]. Among the many evoked arguments [25], is the fact that cancer cells in metastases often develop new molecular alterations under the selective pressure of treatment, immune system, or unfavorable environment [26, 27], suggesting that real-time profiling of the metastasis might be preferable to that of archived tumor. Another argument is the relatively small number of genes tested, with a median number of 209 (range 8–426) in the published studies [6]. Increasing this number might improve the results.

Here, we report the results of the prospective PERMED-01 clinical trial (NCT02342158) that enrolled 550 patients with advanced solid cancers. The main objective was to evaluate the feasibility and clinical impact of a real-time extensive molecular profiling of newly biopsied metastatic sample by using targeted NGS (t-NGS) of the largest gene panel to date and whole-genome array-comparative genomic hybridization (aCGH). We assessed single-gene alterations but also global genomic scores, such as homologous recombination score (HRD) and tumor mutational burden (TMB), and compared the results with those obtained using whole-exome sequencing (WES) in a subset of breast cancer samples.

## Methods

### Study objectives and design

PERMED-01 was a prospective unicentric clinical trial sponsored by and conducted at the Paoli-Calmettes Institute (Marseille, France) (Additional file 1). Detailed information is available in Supplementary Methods (Additional file 2). Its primary objective was to evaluate the number of patients with advanced cancer for whom identification of actionable genetic alterations (AGAs) in tumor samples using t-NGS and aCGH could lead to the delivery of a “matched therapy.” Secondary objectives herein reported included a description of patients with AGA who received a “matched therapy” and their clinical outcome and comparison of AGA identification with t-NGS and aCGH *versus* whole-exome sequencing (WES). Additional secondary objectives that have been or will be reported elsewhere included the description of molecular alterations of advanced solid cancers and their relationship with the clinicopathological characteristics, including progression-free survival and overall survival, their comparison with molecular alterations of the paired primary tumor if available, pan-genomic molecular analysis of metastatic samples with WES [27] and transcriptome analysis, analysis of circulating tumor DNA, analysis of circulating tumor cells (for breast and digestive cancers), and development of preclinical models for prediction/analysis of tumor response/resistance (xenografts, short-term culture, and organoids for breast cancer). Inclusion criteria were age ≥ 18 years, pathological diagnosis of solid cancer, locally advanced or metastatic stage progressive during at least one line of prior therapy and with an accessible lesion for biopsy, Eastern Cooperative Oncology Group (ECOG) Performance Status ≤ 2, affiliation to Social Insurance, and signed informed patient’s consent for participation. Exclusion criteria were symptomatic or progressive leptomeningeal or brain metastases, bone or brain metastasis as sole metastatic site, pregnancy or breastfeeding, and person in an emergency situation or subject to a measure of legal protection or unable to express consent. All patients gave their informed consent for inclusion. Once the patient had been enrolled in the trial, a tumor biopsy or resection was planned. The study was reported according to the CONSORT checklist.

### Biopsy and genome analysis

All genomic analyses were done on de novo tumor biopsies or resections, and not archival samples. Only frozen samples with at least 30% of tumor cells were retained for analysis. Tumor DNA and germline DNA (when available) were extracted and the genomic profiles were established by using aCGH and t-NGS as described [28]. Detailed information is available in Supplementary Methods (Additional file 2). Briefly, aCGH was done onto high-resolution 4 × 180 K CGH microarrays (SurePrint G3 Human CGH Microarray Kit, Agilent Technologies, Massy, France). All probes were mapped according to the hg19/NCBI human genome mapping database. Analysis was limited to the 802 genes present in at least of one NGS panel. The gene copy number was categorized into amplification (Log_2_ratio > 1) or deletion (Log_2_ratio < − 1). For each tumor, a HRD score (HRD_aCGH_ score), based on losses of heterozygosity (LOH), was calculated from all tested aCGH genes [29]: a score ≥ 10 was considered as HRD-high.

Regarding t-NGS, four chronologically extended home-made panels of genes selected for their involvement in cancers were used (Additional file 3: Table S1) covering 395, 494, 560, and 795 genes respectively, and including from 49 to 67 cancer predisposition genes analyzed by the BROCA Cancer Risk panel (https://testguide.labmed.uw.edu/public/view/BROCA). Tumor samples and matched normal samples (available for 315 patients) were sequenced at respective median depths of 732× and 387×. Sequence data were aligned to the human genome (UCSC hg19) and alignment processed as described [30]. The tumor mutational burden (TMB) and MSI-H status were defined in the 295 tumors with a matched normal sample sequenced. The threshold for TMB-high was 10 mutations/Mb [31]. Microsatellite instability detection was done using the software MSIsensor [32] that computes a “MSI score” and a 10% cut-off to detect MSI-H tumors.

WES data were available for 112 pairs of metastatic breast cancer and matched-germline DNA previously profiled using Illumina© technology [27, 33], allowing the comparison of AGAs, HRD score, and TMB. The HRD score was measured from WES data (HRD_WES_) as described [34], by compiling the three independent measures of genomic instability: number of LOH, number of telomeric-allelic imbalances (TAI), and number of large-scale state transitions (LST), scored from FACETS results. The score was the sum of TAI, LST, and LOH scores. The profile was considered as HRD-high when the HRD_WES_ score was ≥ 42 [35]. Regarding the TMB, the comparison was done using both continuous and binary values.

### Molecular precision oncology report and molecular tumor board

Two molecular genomists reviewed all molecular alterations identified and generated a molecular report, which was discussed during our weekly institutional molecular tumor board (MTB) to recommend and prioritize possible matched therapy. The actionability of an alteration was defined by our MTB experts in real time as the existence of a drug targeting the altered protein, either directly or indirectly by impacting the activated pathway. Besides the type of alterations retained according to the type of genes (oncogenes and tumor suppressor genes: see the Supplementary Methods: Additional file 2), the biomarker/treatment association was estimated by using OncoKB [36] (by considering all evidence levels, from 1 to 4) and/or clinical or preclinical data from the literature (suggesting a link with response or resistance) and/or the existence of a clinical trial requiring the alteration for enrollment. AGAs were represented by single-gene alterations, high HRD or TMB, or MSI-H. The recommended therapy was defined as “matched” when its prescription was based upon an AGA identified thanks to the PERMED-01 extensive molecular screening. Otherwise, it was defined as “non-matched therapy.” Of note, this definition was independent from EMA approval at the time of treatment initiation. Treatment assignment was at the discretion of physician and patient. The patients initiating a systemic treatment, “non-matched” or “matched” according to the MTB proposal, were monitored for tumor response. When possible cancer susceptibility was identified, the result was explained to the patient during an oncogenetics consultation.

### Statistical analysis

Detailed information is available in Supplementary Methods (Additional file 2). In order to have a sufficient number of patients with different cancers and with an identifiable AGA, we wanted to evaluate 300 patients enrolled over 3 years. Previous studies [12, 13] had reported a 35% technical failure rate. Thus, we planned to include 460 patients that were enrolled on November 2017. In order to increase certain subpopulations sample sizes, the protocol was amended and a total of 550 patients were enrolled on September 2019. Baseline patient and disease characteristics were summarized using descriptive analysis by using counts and frequencies for categorical variables and medians (ranges) for continuous variables. The primary endpoint was the number of patients with AGAs prospectively identified in real time in tumor samples. A retrospective post hoc analysis of this endpoint was added using a more stringent AGA definition based on the last OncoKB version (v2.10). Secondary endpoints herein reported included a description of clinicopathological characteristics of patients with AGA who received a “matched therapy” *versus* “non-matched therapy,” including their clinical outcome, and a comparison of AGA identification with t-NGS and aCGH *versus* WES. The main efficacy endpoint was the PFS2/PFS1 ratio, defined as the ratio of progression-free survival (PFS2) on treatment given after molecular testing (therapy 2) to the PFS on the immediate previous treatment (PFS1, therapy 1) [8]. A ratio ≥ 1.3 is considered as a non-ambiguous sign of activity for the new treatment, relative to previously received treatments [8]. A post hoc univariate analysis (Fisher’s exact test) searched for clinical parameters associated with ratio ≥ 1.3 among the patients with AGA and treated with “matched therapy” or “non-matched therapy:” three variables were included as continuous variables (patients’ age, number of metastatic sites, and number of previous lines of chemotherapy), whereas other variables were included as categorical variables. Such univariate analysis and the comparison of efficacy endpoints between the “matched therapy” and “non-matched therapy” groups was also done using regression analyses (glm and Cox proportional hazards models) with adjustment upon the cancer type (breast cancer *versus* non-breast cancer) and FDR correction. According to the French law, this interventional research protocol also planned to collect the serious adverse events related to the biological sampling procedures.

## Results

### Study flow and patients’ characteristics

**Fig. 1:**
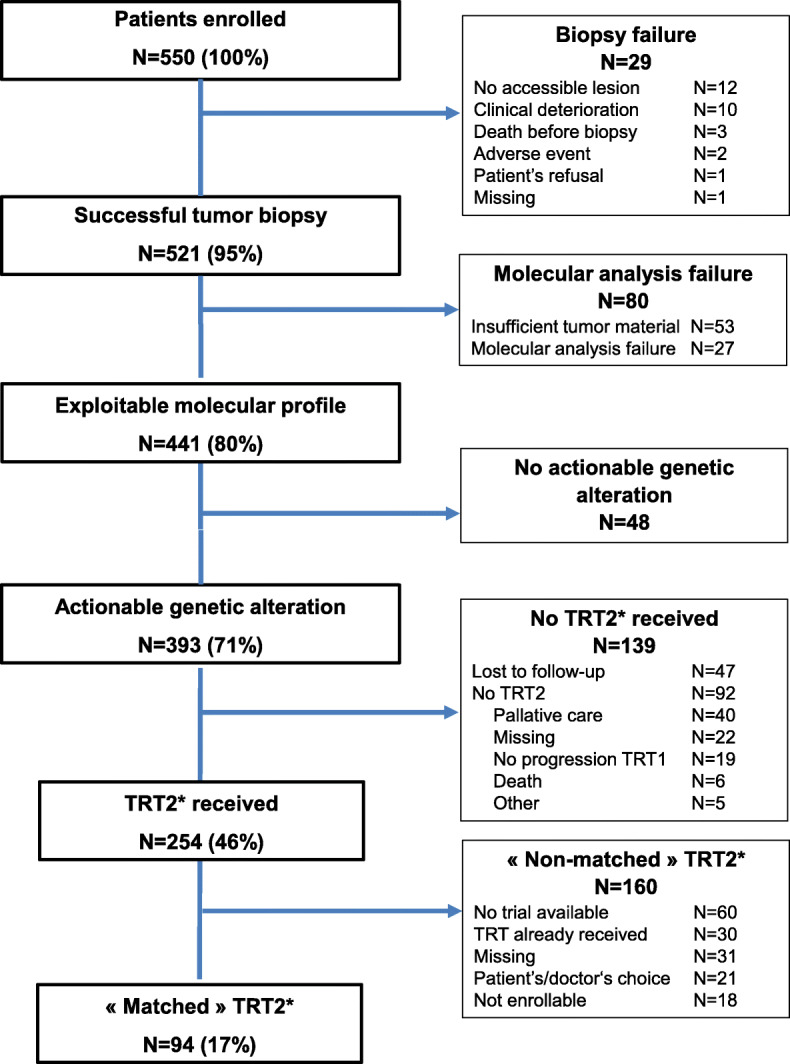
CONSORT diagram. *TRT2, systemic therapy delivered for disease progression after PERMED-01 enrolment

The first patient was enrolled on November 2014. The intermediate analysis after enrolment of the 100th patient showed an 18% technical failure rate, allowing to continue the trial. Until September 2019, we enrolled 550 patients (Fig. 1). Their characteristics are summarized in Table 1. A tumor biopsy was successful in 521 patients (95%). Reasons for failure (*N* = 29) included absence of accessible lesion, patient’s refusal, death, or clinico-biological deterioration. The main sites of biopsy were liver, lymph nodes, then lung. Seven out of 521 patients experienced grade ≥ 2 adverse event within the seven post-biopsy days: atrial fibrillation (grade 2; *N* = 1, liver biopsy), pneumothorax (grade 3; *N* = 2, lung biopsy), fever (grade 3; *N* = 2, lymph node and prostate biopsies), ischemic stroke (grade 5; *N* = 1, patient who had stopped anti-coagulant treatment 3 days before liver biopsy), and acute decompensation of unknown meningeal and brain metastases (grade 5; *N* = 1, lung biopsy). Seven were classified as serious adverse events, requiring hospitalization or prolongation of existing hospitalization: five patients recovered after medical treatment, but two died, including one considered as related to the procedure.

**Table 1:** Patients’ characteristics at inclusion

Groups	[1]	[2]	[3]	[4]	[5]	
Characteristics	Enrolled	Exploitable molecular profile	With AGA	With AGA and “matched therapy”	With AGA and “non-matched therapy”	***p*** value^a^
	***N*** = 550 (100%)	***N*** = 441 (80%)	***N*** = 393 (71%)	***N*** = 94 (17%)	***N*** = 160 (29%)	
**Age, years**						1.47E**−**04
Median (range)	59 (20–84)	59 (20–84)	59 (21–83)	62 (26–83)	56 (22–81)	
**Sex**						0.749
Male	135 (25%)	107 (24%)	92 (23%)	21 (22%)	32 (20%)	
Female	415 (75%)	334 (76%)	301 (77%)	73 (78%)	128 (80%)	
**ECOG performance status**						0.720
0	181 (39%)	148 (39%)	127 (37%)	31 (37%)	55 (42%)	
1	235 (50%)	199 (52%)	179 (52%)	43 (51%)	60 (46%)	
2	52 (11%)	37 (10%)	35 (10%)	10 (12%)	15 (12%)	
Missing	82	57	52	10	30	
**Cancer type**						4.74E**−**02
Breast	268 (49%)	216 (49%)	197 (50%)	42 (45%)	93 (58%)	
Lung	43 (8%)	34 (8%)	29 (7%)	6 (6%)	12 (8%)	
Prostate	39 (7%)	29 (7%)	25 (6%)	10 (11%)	4 (2%)	
Ovary	33 (6%)	30 (7%)	23 (6%)	8 (9%)	10 (6%)	
Pancreas	29 (5%)	22 (5%)	22 (6%)	5 (5%)	2 (1%)	
Colorectal	22 (4%)	21 (5%)	19 (5%)	3 (3%)	10 (6%)	
Sarcoma	18 (3%)	15 (3%)	13 (3%)	3 (3%)	4 (2%)	
Endometrial	17 (3%)	13 (3%)	12 (3%)	5 (5%)	1 (1%)	
Uterine cervix	14 (3%)	10 (2%)	10 (3%)	2 (2%)	4 (2%)	
Liver-biliary tractus	13 (2%)	10 (2%)	10 (3%)	3 (3%)	5 (3%)	
Bladder-ureter	12 (2%)	10 (2%)	7 (2%)	2 (2%)	2 (1%)	
Kidney	9 (2%)	6 (1%)	3 (1%)	0 (0%)	3 (2%)	
CUP	7 (1%)	5 (1%)	5 (1%)	1 (1%)	3 (2%)	
Other	26 (5%)	20 (5%)	18 (5%)	4 (4%)	7 (4%)	
**Site of the biopsy**						0.078
Liver	213 (41%)	188 (43%)	171 (44%)	34 (37%)	69 (43%)	
Lymph node	86 (17%)	67 (16%)	57 (15%)	18 (19%)	22 (14%)	
Lung	61 (13%)	50 (11%)	44 (11%)	9 (10%)	22 (14%)	
Breast	25 (5%)	23 (5%)	19 (5%)	4 (4%)	6 (4%)	
Peritoneum	23 (4%)	21 (5%)	18 (5%)	2 (2%)	10 (6%)	
Skin	22 (4%)	19 (4%)	18 (5%)	3 (3%)	10 (6%)	
Prostate	10 (2%)	6 (1%)	5 (1%)	3 (3%)	1 (1%)	
Pancreas	6 (1%)	4 (1%)	4 (1%)	2 (2%)	0 (0%)	
Pleura	6 (1%)	3 (1%)	3 (1%)	0 (0%)	3 (2%)	
Colorectal	2 (0%)	2 (0%)	2 (1%)	1 (1%)	1 (1%)	
Other	60 (12%)	56 (13%)	50 (13%)	17 (18%)	16 (10%)	
Missing	36	2	2	1	0	
**Pathological type**						0.788
Carcinoma	522 (94%)	418 (95%)	374 (95%)	91 (97%)	151 (94%)	
Sarcoma	18 (3%)	15 (3%)	13 (3%)	3 (3%)	4 (2%)	
Germ cell tumor	3 (1%)	3 (1%)	2 (1%)	0 (0%)	2 (1%)	
Melanoma	3 (1%)	3 (1%)	3 (1%)	0 (0%)	2 (1%)	
Other	4 (1%)	2 (0%)	1 (0%)	0 (0%)	1 (1%)	
**Extension stage**						0.506
Metastatic	520 (96%)	422 (96%)	376 (96%)	88 (95%)	154 (97%)	
Locally advanced	24 (4%)	17 (4%)	15 (4%)	5 (5%)	5 (3%)	
Missing	6	2	2	1	1	
**Number of metastatic sites**						0.932
Median	2	2	2	2	2	
Range	0 to 7	0 to 7	0 to 7	0 to 7	0 to 7	
**Number of previous lines of chemotherapy for advanced disease**						0.075
Median	3	3	3	3	2	
Range	1 to 12	1 to 12	1 to 12	1 to 12	1 to 12	
Missing	78	57	53	6	29	

^a^Comparison between groups [4, 5]: Mann-Whitney test for continuous variables, Fisher’s exact test for categorical variables

### Somatic molecular alterations

An exploitable molecular profile (aCGH and t-NGS) was obtained in 441 patients (80% out of 550). The reasons for “molecular failure” (*N* = 80) included insufficient quantity and/or quality of tumor material (*N* = 53), and experimental failure (*N* = 27) (Fig. 1). The median time from inclusion to discussion in MTB was 58 days (range, 1–645). The characteristics of patients with exploitable profile (Table 1) were similar to those of the entire cohort. The five most frequent cancer types were breast (*N* = 216), lung (*N* = 34), ovary (*N* = 30), prostate (*N* = 29), and pancreas (*N* = 22) carcinomas (Additional file 4: Fig. S1). The most frequent pathological type was carcinoma. The advanced disease mainly corresponded to metastatic disease (96%). The median number of different metastatic sites was 2 (range, 0–7), and the median number of prior chemotherapy lines for advanced disease was 3 (range, 1–12).

Among the 441 exploitable profiles [37, 38], 6336 somatic gene alterations were found (Additional file 4: Fig. S2 and Fig. S3), including 5056 mutations and 1280 copy number alterations (CNAs: 678 deletions, 602 amplifications). The median number of alterations per patient was 12 (range, 0–108). The 10 most frequently altered genes were *TP53* (52%), *PIK3CA* (20%), *NEB* (16%), *USH2A* (16%), *KRAS* (14%), *CSMD3* (13%), *LRP1B* (13%), *ESR1* (11%), *DMD* (11%), and *ATM* (10%). A high HRD_aCGH_ score was observed in 182 patients (Additional file 4: Fig. S4a). Pancreas, ovary, colorectal, breast, and prostate carcinomas had the highest percentage of high score. A positive correlation existed between the HRD_aCGH_ score and the presence/absence of mono- and bi-allelic pathogenic alterations of genes involved in homologous recombination, such as *BRCA1/2* (*p* = 3.40E-10, Kruskal-Wallis test; Additional file 4: Fig. S4b). High TMB was observed in 22 out of 293 informative patients (Additional file 4: Fig. S4c) and was higher in lung cancer than in breast, ovary, prostate, and pancreatic carcinomas (*p* = 2.15E-03, Kruskal-Wallis test). Four out of 287 informative patients displayed MSI-H status, including three with high TMB.

### Actionable somatic molecular alterations

**Fig. 2:**
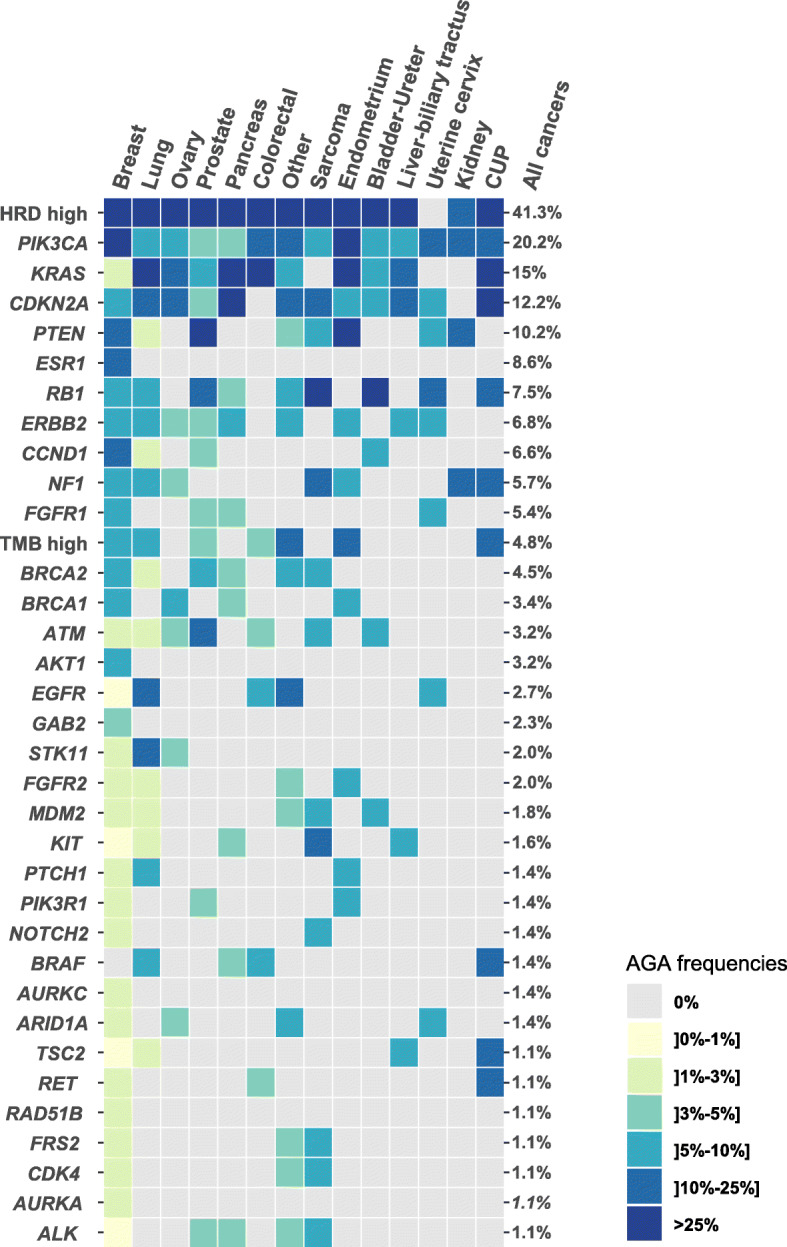
List and incidence of AGAs. The top 35 AGAs identified in more than 1% of 441 exploitable samples are ordered from top to bottom according to the decreasing percentage of altered patients in the whole population with exploitable profile (% to the right of color matrix). The cancer types are ordered from left to right according to the number of samples with alterations for those 35 AGAs. The percentage of patients with each AGA *per* cancer type is color-coded as indicated to the right of matrix

Through the 441 exploitable samples, 952 AGAs were identified in real time by our MTB, and 393 patients (71% (two-sided 90%CI 68–75) of 550 enrolled patients) displayed at least one AGA. Their characteristics are summarized in Table 1. The median number of AGAs per patient was 2 (range, 0–8). AGAs included 744 single-gene alterations comprising 477 mutations and 267 CNAs and concerning 95 genes, and 208 global genomic scores, mainly represented by high HRD (*N* = 182), then high TMB (*N* = 22). Figure 2 shows the top 35 AGAS. High HRD was by far the most frequent (41% of patients), followed by alterations of *PIK3CA* (20%), *KRAS* (15%), *CDKN2A* (12%), *PTEN* (10%), *ESR1* (9%), *RB1*, *ERBB2,* and *CCND1* (7%), and *NF1* (6%). High TMB was observed in 5% of samples. Although frequent, a high HRD score alone accounted for only 4% of patients: exclusion of this score let 67% of patients with at least one AGA. Among the top 35 AGAS, there was an overrepresentation of genes involved in the PIK3/AKT/MTOR pathway, DNA repair, and cell cycle.

### Patients treated with “matched therapy”

Within the 393 patients with AGA, 254 received a systemic therapy for progression after inclusion, including 94 patients who received a “matched therapy” (Fig. 1).

**Fig. 3:**
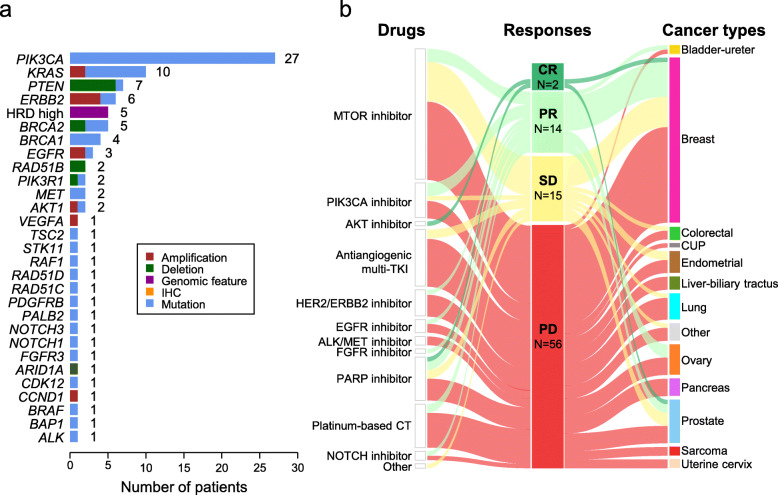
“Matched therapies:” list of AGAs and therapeutic responses. **a** AGAs targeted by “matched therapies.” Seven patients with mutations in homologous recombination (HR) DNA repair-related genes displayed also high HRD score (*BRCA1*: 1; *BRCA2*: 3; *RAD51B*: 2; *RAD51D*: 1). **b** Objective responses to “matched therapies” displayed as alluvial plots. Left: therapies ordered by drug classes. Middle: responses ordered from CR to PD. CR = complete responses, PR = partial responses; SD = stable disease, and PD = progressive disease. Right: cancer types alphabetically ordered

These 94 patients represented 17% (95%CI 14-21) of the 550 enrolled patients (Table 1 and Additional file 3: Table S2). They had received a median of 3 (range, 1–12) prior lines of chemotherapy for advanced disease. The corresponding AGAs were identified using NGS (*N* = 61), aCGH (*N* = 28), and both aCGH and NGS (*N* = 5), and concerned 28 genes and one genomic score. The most frequent AGAs included *PIK3CA* mutations (*N* = 27), *KRAS* mutations/amplifications (*N* = 10), *PTEN* deletions/mutations (*N* = 8), *ERBB2* amplifications/mutations (*N* = 6), *BRCA2* mutations (*N* = 6), and high HRD score (*N* = 5) (Fig. 3a). The “matched therapies” are summarized in Table 2 and detailed in Table S2 (Additional file 3). The most frequent ones were PIK3/AKT/MTOR inhibitors. Eighty-four patients were treated with MTT (single-agent in 71, combination in 13), 10 were treated with platinum-based chemotherapy (single-agent in 8, combination in 2), and one with PD1-inhibitor (in combination with MTT). Seventy-eight percent of patients were treated within phase I/II trials.

**Table 2:**
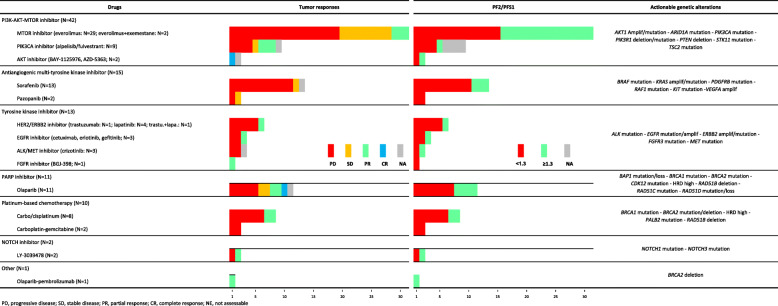
AGA-“matched therapies” delivered to the 94 patients, clinical outcome, and AGAs

The main reasons for giving “non-matched therapy” after biopsy to the other 160 patients with an AGA were as follows: no trial available, therapy already received during the previous lines, and patients’ or physicians’ choice (Fig. 1**)**. The remaining 139 patients with AGA did not receive any therapy after the biopsy for the following reasons (Fig. 1): lost to follow-up (*N* = 47), and no further therapy for different causes (*N* = 92), including mainly palliative care or death.

### Clinical efficacy of “matched therapy”

**Fig. 4:**
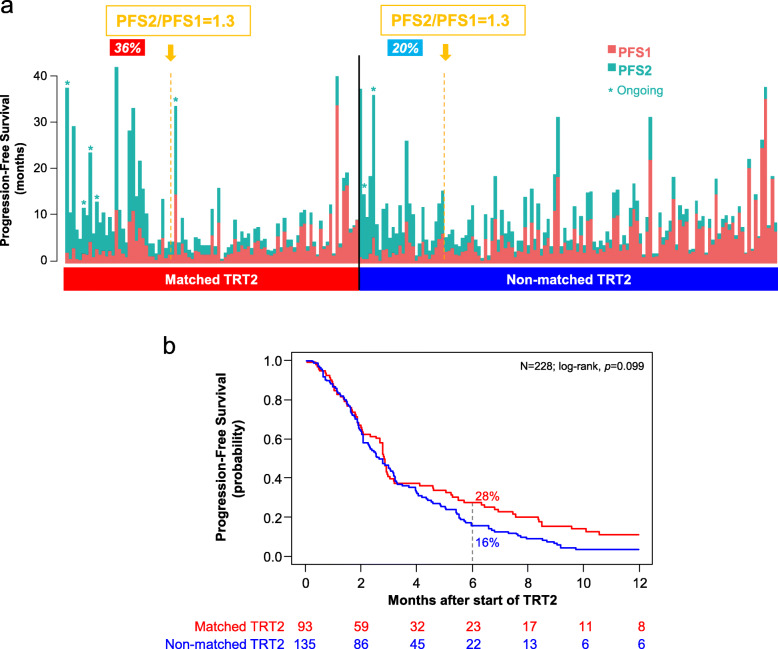
PFS in patients with AGA treated with a “matched therapy” *versus* a “non-matched therapy.” **a** PFS2 and PFS1 durations in the two groups of patients with AGA and treated with “matched therapy” (left) and with “non-matched therapy” (right). The patients are ordered from left to right by decreasing PFS2/PFS1 ratio. The vertical dashed orange lines indicate the ratios = 1.3: patients to the left have a ratio ≥ 1.3. **b** Kaplan-Meier curve of PFS2 in patients treated with “matched therapy” (red curve) and in patients treated with “non-matched therapy” (blue curve)

Among the 94 patients with “matched therapy” (Additional file 3: Table S2), the median PFS2/PFS1 ratio was 0.91 (range, 0.0–14.9), and 32 out of 89 informative patients (36%, 95%CI 26–47) had a ratio ≥ 1.3 (Table 3, Fig. 4a). None of the tested parameters was significantly associated with a ratio ≥ 1.3 (Additional file 3: Table S3). The PFS2/PFS1 ratio was associated with OS, with a hazard ratio (HR) for death equal to 0.51 (95%CI, 0.30–0.87) in case of ratio ≥ *versus* < 1.3 (*p* = 1.31E−02, Wald test). The median PFS2 was 2.9 months (95%CI 2.7–3.2) and the 6-month PFS2 was 28% (95%CI 20–39) (Fig. 4b). The response rates (Table 2, Fig. 3b) were 2% for complete responses (CR: olaparib for *BRCA2* mutation and high HRD score in prostate cancer, and capivasertib for *PIK3CA* mutation in breast cancer), 17% for partial responses (PR), 16% for stable disease (SD), and 65% for progressive disease (PD). The objective response rate was 19% (95%CI 12–29) and disease control (DC) rate (CR + PR + SD) was 35% (95%CI 25–46). The 6-month PFS2 was 79% (95%CI 66–95) in patients with DC *versus* 2% (95%CI 0–12) in patients without (*p* = 5.11E–15), with a HR for PFS event equal to 8.6 (95%CI 4.8–15.2) between the two groups. The median OS was 8.1 months (95%CI 6.2–12.2) and the 6-month OS was 62% (95%CI 52–73).

**Table 3:** Efficacy parameters in the two groups of patients with AGA treated with “matched therapy” *versus* “non-matched therapy”

	Matched therapy		Non-matched therapy		***p*** values^b^
	***N*** = 94		***N*** = 160		
	*N*	% ^a^	*N*	% ^a^	
**PFS2/PFS1**					
Median (range)	0.91 (0–14.9)		0.67 (0–25.3)		
*N* with ratio ≥ 1.3 (95%CI)	32	36% (26–47)	26	20% (14–29)	**0.013**
Missing	5		33		
**PFS2**					
Median, months (95%CI)	2.9 (2.7–3.2)		2.8 (2.2–3.2)		
Missing	1		25		
6-month PFS2 (95%CI)	28% (20–39)		16% (11–24)		0.099
**Clinical response**					
Complete response	2	2%	2	1%	0.249
Partial response	15	17%	33	24%	
Stable disease	14	16%	12	9%	
Progressive disease (PD)	58	65%	93	66%	
Disease control (DC) (95%CI)	31	35% (25–46)	47	34% (26–42)	0.887
NA	5		20		
**PFS2 if DC**					
Median, months (95%CI)	8.5 (6.9–19.8)		5.7 (5.4–7.3)		
6-month PFS2 (95%CI)	79% (66–95)		41% (29–58)		**1.86E−03**
Missing	0		3		
**PFS2 if PD**					
Median, months (95%CI)	1.9 (1.6–2.8)		1.9 (1.6–2.1)		
6-month PFS2 (95%CI)	2% (0–12)		1% (0–8)		0.824
Missing	0		7		
**OS**					
Median, months (95%CI)	8.1 (6.2–12.2)		8.9 (6.5–11.1)		
6-month OS (95%CI)	62% (52–73)		59% (51–68)		0.791
Missing	3		11		

^a^% of informative cases; *NA* not assessable; ^b^Fisher’s exact test for categorical variables, log-rank test for 6-month survivals

We compared these endpoints to those of the 160 patients with AGA treated with “non-matched therapy.” Clinical characteristics were similar between both groups, except age (younger in the “non-matched therapy” group, *p* = 1.47E−04) and cancer type (more breast and colorectal cancers in the “non-matched therapy” group and less prostate, pancreas, and endometrial cancers, *p* = 4.74E−02) (Table 1). In the “non-matched therapy” group, the median PFS2/PFS1 ratio (0.67; range, 0–25.3) and the percentage of patients with ratio ≥ 1.3 (20% [95%CI 14–29]) were significantly inferior to those observed in the “matched therapy” group (*p* = 1.30E−02, Fisher exact test; Table 3, Fig. 4a). The median PFS2 was 2.8 months (95%CI 2.2–3.2) and the 6-month estimated PFS2 was 16% (95%CI 11–24) (Fig. 4b), similar to the “matched therapy” group, as was the DC rate (34% [95%CI 26-42], *p* = 0.887, Fisher exact test). In patients with DC, the 41% (95%CI 29–58) 6-month PFS2 was significantly inferior to that observed in the “matched therapy” group (*p* = 1.86E−03, log-rank test; Table 3), and the HR for PFS event between “non-matched therapy” patients with *versus* without DC (6.2 [95%CI 4.0–9.5]) tended to be inferior to that observed in the “matched therapy” group. These significant differences between the “matched therapy” and “non-matched therapy” groups remained significant in regression analyses after adjustment upon the cancer type (breast cancer *versus* non-breast cancer) and after FDR correction (Additional file 3: Table S4). The 6-month OS did not differ from that of the “matched therapy” group.

Finally, in a post hoc analysis, we searched for variables predictive for PFS2/PFS1 ratio ≥ 1.3 in the pooled population of patients with AGA treated with “matched therapy” or “non-matched therapy” (Table 4). Only the use of “matched therapy” was significantly associated with an improved ratio (*p* = 1.30E−02, Fisher’s exact test). In regression analysis, “matched therapy” remained significant after adjustment upon the cancer type without and with FDR correction (*p* = 9.83E−03 and *q* = 8.85E-02, glm) (Additional file 3: Table S5).

**Table 4:** Univariate analysis for PFS2/PFS1 ratio ≥ 1.3 in treated patients with AGA

Characteristics		PFS2/PFS1		***p*** values^a^
	***N***	< 1.3 (***N*** = 158)	≥ 1.3 (***N*** = 58)	
**Age at inclusion, years**				
Median (range)	216	57 (30–83)	61 (22–79)	0.426
**Sex**				0.583
Male	47	36 (23%)	11 (19%)	
Female	169	122 (77%)	47 (81%)	
**ECOG performance status at inclusion**				0.179
0	72	49 (36%)	23 (48%)	
1	88	66 (49%)	22 (46%)	
2	23	20 (15%)	3 (6%)	
Missing	33			
**Cancer type**				0.869
Breast	111	80 (51%)	31 (53%)	
Lung	16	12 (8%)	4 (7%)	
Prostate	14	8 (5%)	6 (10%)	
Ovary	16	10 (6%)	6 (10%)	
Pancreas	4	3 (2%)	1 (2%)	
Colorectal	11	10 (6%)	1 (2%)	
Sarcoma	6	5 (3%)	1 (2%)	
Endometrial	5	3 (2%)	2 (3%)	
Uterine cervix	6	4 (3%)	2 (3%)	
Liver-biliary tractus	6	5 (3%)	1 (2%)	
Bladder-ureter	4	4 (3%)	0 (0%)	
Kidney	3	3 (2%)	0 (0%)	
CUP	4	3 (2%)	1 (2%)	
Other	10	8 (5%)	2 (3%)	
**Pathological type**				1.000
Carcinoma	206	149 (94%)	57 (98%)	
Sarcoma	6	5 (3%)	1 (2%)	
Melanoma	2	2 (1%)	0 (0%)	
Germinal tumor	2	2 (1%)	0 (0%)	
**Extension stage at inclusion**				0.450
Metastatic	205	149 (95%)	56 (98%)	
Locally advanced	9	8 (5%)	1 (2%)	
Missing	2			
**Number of metastatic sites**				
Median (range)	216	2 (0–6)	2 (0–7)	0.568
**Number of previous lines of chemotherapy for advanced disease**				
Median (range)	185	3 (1–12)	3 (1–12)	0.671
Missing	31			
**Type of therapy**				**1.30E−02**
Matched	89	57 (36%)	32 (55%)	
Non-matched	127	101 (64%)	26 (45%)	

^a^Mann-Whitney test for continuous variables, Fisher’s exact test for categorical variables

### AGA identification with WES *versus* t-NGS/aCGH

For 112 patients with breast cancer, we could compare the results of AGA identification between WES and t-NGS/aCGH. In 10 patients, no AGA was retained with both approaches. Among the 102 remaining patients, t-NGS/aCGH identified 284 AGAs, including 226 single-gene alterations (137 mutations, 89 CNAs; 62 genes), and 58 genomic scores (50 high HRD, 7 high TMB, 1 MSI-H), whereas WES identified 225 AGAs, including 186 single-gene alterations (121 mutations, 65 CNAs; 52 genes), and 39 genomic scores (35 high HRD, 4 high TMB).

**Fig. 5:**
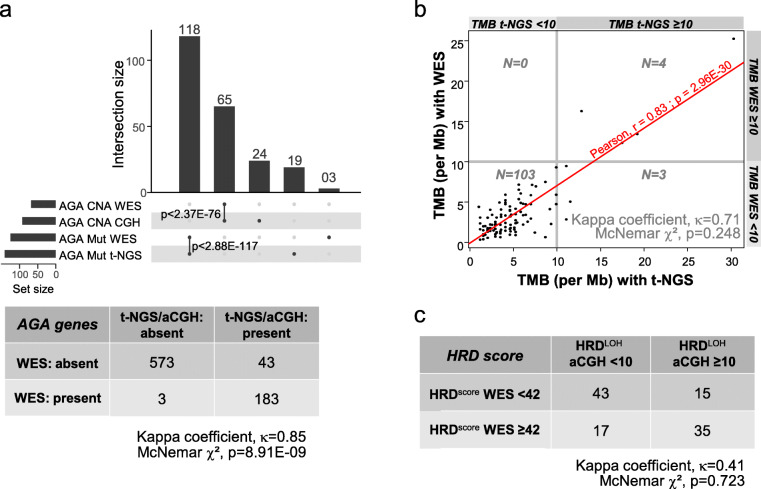
Comparison of AGAs identified using WES *versus* t-NGS/aCGH. **a** *Top*: upSet charts showing the comparison of single-gene AGAs identified by WES *versus* t-NGS/aCGH approaches in 112 patients with advanced breast cancer; *bottom*: cross-table. **b** Comparison of TMB analyzed as continuous value and as binary value. **c** Comparison of HRD score using the LOH-based HRD_aCGH_ score *versus* the HRD_WES_ score

Within the 802 genes tested, 98% (183 out of 186) of single-gene AGAs identified using WES were also found using t-NGS/aCGH (Fig. 5a), including all CNAs and all but three mutations (2 *PTEN* frameshift*,* 1 *BRCA2* frameshift mutations). By contrast, 43 single-gene alterations (24 CNAs, 19 mutations) identified using t-NGS/aCGH were not identified using WES. The global concordance was high (94%) with Kappa coefficient *κ* equal to 0.85. The McNemar *χ*^2^ test was significant (*p* = 8.91E−09), meaning a significant effect of the sequencing type on AGA detection, with higher sensitivity for t-NGS/aCGH (98%) than WES (81%), and lesser specificity (93% *versus* 99%).

There was a strong correlation between t-NGS and WES for TMB (Fig. 5b), as continuous value (*R* = 0.83; *p* = 2.95E−30) and as binary value with strong concordance (Kappa coefficient *κ* = 0.71); the type of test did not impact the TMB status (*p* = 0.248, McNemar *χ*^2^ test). Similar observation was done for the HRD scores defined by aCGH (LOH-based HRD_aCGH_) *versus* WES (HRD_WES_) (Kappa coefficient *κ* = 0.41; *p* = 0.723, McNemar *χ*^2^ test; Fig. 5c).

Finally, 95% of patients displayed at least one AGA concordant with both approaches, including 16 out of 19 patients (84%) treated with matched therapy. Among the three discordant treated patients who would have not displayed AGA by WES analysis (*EGFR* amplification, high HRD, *PTEN* deletion), two had benefited from “matched therapy” (cetuximab and everolimus) with improved PFS2/PFS1 ratio.

### Germline molecular alterations

**Fig. 6:**
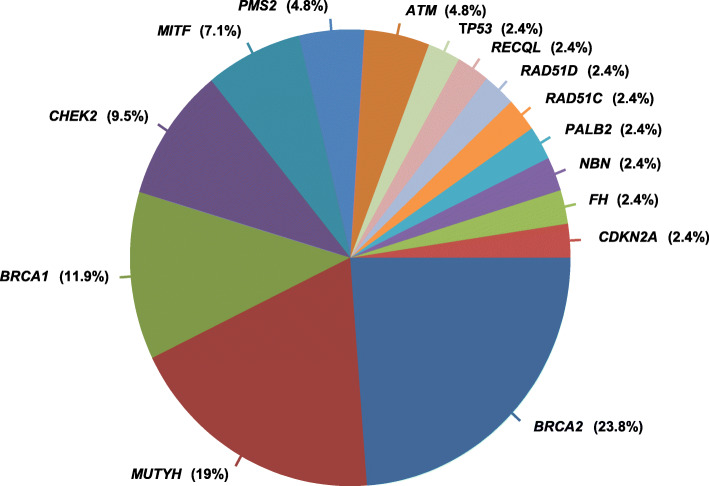
Genes with pathogenic germline variants. For each gene, the percentage of PGVs is calculated from the total number of 42 PGVs

Germline DNA sequencing was available for 295 patients. Analysis was limited to the 63 cancer predisposition genes of the BROCA Cancer Risk panel. We identified 2006 germline variants (GVs), 42 of which were pathogenic or likely pathogenic (PGVs) [39]. PGVs were identified in 39 patients (13%, 95%CI 9.6–17.6) and targeted 15 genes (Fig. 6, and Additional file 3: Table S6). They corresponded to 15 nonsense mutations, 14 deleterious missense mutations, 9 frameshift mutations, and 4 deleterious splice site mutations. The 15 genes corresponded to either predisposition genes concordant with the patient’s pathology (high-risk genes (e.g. *BRCA1*, *BRCA2*, *PALB2*), or medium/low-risk genes with no specific management recommendation in France (*ATM*, *CHEK2*, *RECQL*)), or genes predisposing for pathologies different from the patient’s pathology (incidental discoveries: *CDKN2A*, *FH*, *MITF*, *MUTYH*) [37]. More than 85% of the PGVs were observed in genes related to DNA repair, of which the three most frequent were *BRCA2* (22%), *MUTYH* (19.5%), and *BRCA1* (12.2%). Twelve out of 39 (31%) patients with PGV displayed a somatic alteration (loss, LOH) in the tumor DNA.

The results were explained to the patient by an oncogeneticist to offer adapted follow-up. Based on their personal and/or familial history (Additional file 3: Table S6), 24 patients (7% of 295 tested patients; 62% of 39 patients with PGVs) had been referred to oncogenetics consultation before inclusion in PERMED-01, leading to the prior identification of loss-of-function PGVs in *BRCA1* (*N* = 5) and *BRCA2* (*N* = 9) in 14 patients. The mutated genes identified in the ten other patients (*ATM, CDKN2A, CHEK2, FH, MUTYH, RAD51D,* and *RECQL*) had not been previously analyzed due to the absence of recommendation for sequencing at the time of oncogenetics consultation (*RAD51D*) and/or for clinical management in breast and/or ovarian cancer predisposition (other genes). An oncogenetics consultation was recommended for 19 patients (first consultation in 10, and second in 9) with PGVs after PERMED-01 sequencing (6% of tested patients; 49% of patients with PGVs), including 11 after identification of incidental PGVs in genes with clinical management recommendations in another pathology (8 monoallelic PGVs in *MUTYH*, 2 in *PMS2*, and 1 in *FH*) and 5 after identification of PGVs in genes related to the pathology (one patient with PGV in *BRCA1*, *PALB2*, *RAD51C*, *RAD51D*, and *TP53*). Only five consultations could be carried out (26.3%), because of the patient’s death in more than 50% of the cases.

## Discussion

We prospectively enrolled 550 patients with advanced solid cancers in PERMED-01: based on our AGA definition, 71% displayed at least one AGA in the newly biopsied tumor sample, and 17% received a “matched therapy” on progression, which provided a PFS2/PFS1 ratio ≥ 1.3 in 36% of cases, representing 6% of enrolled patients. For comparison, 20% of patients with AGA and “non-matched therapy” displayed a ratio ≥ 1.3. Within all patients with AGA treated on progression, the use of “matched therapy” was the sole variable associated with an improved ratio.

Comparatively to other precision medicine trials, we included two design modifications susceptible to improve the results. First, given the known evolution of tumor genome with time, we used only new biopsies done inside the trial, rather than archival samples. Eighty percent of enrolled patients displayed an exploitable molecular profile within a median time of ~ 2 months after enrolment, confirming the feasibility. Most of genomics failures came from insufficient quantity and/or quality of biopsied material. The safety of biopsy was reported. Among the two deaths occurring within the post-biopsy week, one was related to biopsy (ischemic stroke due to erroneous handling of anti-coagulant drugs by the patient). Second, we increased the number of genes tested and analyzed not only single-gene alterations, but also clinically relevant genomic scores.

An AGA was identified in 71% (two-sided 90%CI 68–75%) of enrolled patients. Based on our literature review, this percentage may appear higher than previous studies, which reported a median of 42% (95%CI 40–63) [9, 10, 14–18, 40, 41]. With the 300-sample size initially chosen, there was a power exceeding 95% to demonstrate that this rate was superior to one of the highest reported in the literature, e.g., 60%, if assuming a type I error of 5%, expecting a 10% benefit, e.g., a desirable AGA identification rate of 70%, and using an exact right-sided binomial test. This test turned out to be highly significant in our study (*p* < 0.001). However, we acknowledge that every inter-study comparison is difficult because of many divergences, including not only different cancer types enrolled, different gene panels and profiling techniques, but also different AGA definitions,…. For example, the higher number of “candidate genes” tested—≥ 494 in 94% of our exploitable profiles *versus* a median of 209 genes (range, 8 to 426) in the published studies [6]—might be an explanation for higher AGA percentage, although this remains to be proven as discussed below. One major explanation likely lies on our AGA definition, less stringent than those used through literature. Our MTB experts had defined the “actionability” in real time by using all OncoKB evidence levels (from 1 to 4) and/or clinical/preclinical studies supporting a biomarker treatment association in terms of response and/or resistance. Furthermore, we considered not only the single-gene alterations, but also the genomic scores (HRD-high, high TMB, and MSI-H status) not included in the previous studies. This AGA definition is a critical issue of precision medicine that calls for caution in inter-study comparison. Several definitions are present through the literature explaining in part the variable reported percentages of patients with AGA. Recently, the ESMO Precision Medicine Working Group recommended “that genomic reports include the ranking of the genomic alterations either by ESCAT or OncoKB” [42]. A retrospective post hoc analysis of our data using a more stringent AGA definition based on the last OncoKB version (v2.10) identified 53% of patients with Level 1–4 AGAs and 38% with Levels 1–3. This percentage is close to the 42% median found in literature and similar to the 37% reported in the large MSKCC study that considered Levels 1–3 [41]. Thus, depending on the definition, our percentage of patients with AGA decreased from 71 to 67% after exclusion of HRD scores, to 53% by considering only OncoKB Levels 1–4 and 38% for Levels 1–3. Thus, our AGA definition likely counts for much of the high 71% percentage of patients we found, making every inter-study comparison difficult. However, an additional explanation for this percentage may be the profiling of metastatic samples (*versus* primary tumors) and the prominence of breast cancers (*versus* non-breast cancers), two factors reported as associated with a higher number of molecular alterations that correlated with a higher number of AGAs [21].

A high HRD score was observed in 41% of patients, a level compatible with the rates observed in a series of 5203 solid primary cancers [34] (35% when applied to the whole series, and 43% when applied to breast, lung, ovary, prostate, and colorectal cancers, the five cancer types prominent in our series; Additional file 3: Table S7), and the known higher frequency of HRD in metastases than in primary tumors [28, 43, 44]. Thus, one bias in enrollment (prominence of certain cancer types) and one selection criterion (metastatic samples) of our study might explain such enrichment for HRD-positive tumors. High HRD score was associated with pathogenic alterations of genes involved in homologous recombination, but no DNA alteration was found in several cases, suggesting alternative mechanisms for HRD not detectable using sequencing alone and usefulness of HRD score.

Despite this 71% rate, only 17% of enrolled patients (23% of patients with AGAs) received a “matched therapy” for disease progression, in agreement with the 8–27% (median 15.5%) rates reported in other trials based on aCGH and/or t-NGS [9, 10, 14–18, 21, 40, 45]. Ninety-five percent of AGAs leading to “matched therapy” were single-gene alterations (65% were represented by six genes: *PIK3CA*, *KRAS*, *PTEN*, *ERBB2*, and *BRCA1/2*), whereas 5% were high HRD score. This relatively low rate of patients treated with “matched therapy” has several explanations more or less interrelated: difficulty of drug access within and outside clinical trials, end-stage patients not enrollable in clinical trials, and lack of clinical evidence sufficiently convincing for the physician. Nevertheless, the 160 patients with AGA who received a “non-matched therapy” served as an interesting control group, which could minimize any group biases.

Today, the clinical benefit of “genomics-matched therapy” remains not proven [6]. The first precision medicine trial was reported in 2010 [8]. The expression of 61 target genes/proteins was measured in tumors from 86 patients with refractory metastatic cancer: 66 patients received a matched therapy, of which 27% displayed a PFS2/PFS1 ratio ≥ 1.3. The subsequent retrospective or prospective studies reported conflicting results [9, 10, 14–18, 21, 23, 40, 41, 44]. The only randomized study published to date [45] failed to show improvement in PFS between the precision medicine *versus* standard-of-care arms. Several reasons were evoked including the limited number and old-generation character of 11 available targeted therapies and the heavily pretreated nature of patients. In contrast to this negative study, several non-randomized studies suggested a benefit. Like these latter studies, PERMED-01 was a non-randomized trial, not designed to compare the clinical efficacy of “matched therapy” versus that of “non-matched therapy”. Thus, the results require cautious interpretation, but interesting observations could be done.

A key finding was that 36% of patients who received a “matched therapy” displayed better outcome than with their previous therapy (PFS2/PFS1 ratio ≥ 1.3), *versus* 20% of patients with AGA treated with “non-matched therapy.” This percentage of patients treated with “matched therapy” and displaying a ratio ≥ 1.3 was 33% in MOSCATO [15], and 28% in the Institut Bergonié’s study [14]. These percentages in patients treated with “matched therapy” and “non-matched therapy” were 25% and 26% respectively in EXOMA study [16], but 45.3% and 19.3% in PREDICT [21]. The identification of factors predictive for ratio ≥ 1.3 might help identify the candidates to “matched therapy.” In our series, and as reported [15, 16], no tested variable, including the drug class and the evidence level of AGA, was associated with benefit, but the number of samples was small, and such post hoc analysis should be considered only as hypothesis generating. Although this ratio was associated with OS, OS was not different between our two groups of patients with AGA. Higher response rates, PFS, and/or OS in the “matched” *versus* “non-matched” groups have been reported in non-randomized trials enrolling patients with multiple cancer types [9, 10, 18, 21, 23] or with specific cancers such as pancreas [19, 46], lung [12, 47, 48], gastric [49] carcinomas, and in meta-analyses of phase I-II trials [50, 51]. In our series, the rates of clinical responses and DC in these patients with refractory disease were interesting (19% and 35% in the “matched therapy” group), but not different from those observed in the “non-matched therapy” group. However, the duration of DC (SD + PR + CR) was longer in the “matched therapy” group, confirming the IMPACT study [10, 18], in which the difference in median PFS and OS between responders and non-responders was more pronounced in the “matched” group than the “non-matched” group. Within all patients with AGA treated on progression, the use of “matched therapy” was the sole variable associated with an improved ratio. Interestingly, all these significant efficacy differences in favor of the “matched therapy” group persisted after adjustment upon the cancer type (breast cancer *versus* non-breast cancer) and after FDR correction. Of course, only randomized trials, such as ongoing SAFIR02 (NCT02117167) or IMPACT2 (NCT02152254), will be able to demonstrate the actual benefit of precision medicine.

Our results suggest a few roads for improvement in front of the hurdles encountered in precision medicine [6]. One of the proposed solutions is to increase the number of identified AGAs. In this context, our comparison on 112 metastatic breast cancers suggests that WES does not provide benefit when compared with t-NGS/aCGH applied to a ~ 500-gene panel. Lesser sensitivity in term of single-gene AGAs was observed with WES, likely because of lower sequencing depth, and 16% of patients effectively treated with “matched therapy” would have not been treated using WES data. Interestingly, we showed the type of test (WES *versus* t-NGS/aCGH) did not impact the TMB and HRD statutes, suggesting the reliability of t-NGS/aCGH for assessing these relevant markers. To our knowledge, such comparison on the same samples has never been reported in the literature. Conversely, we retrospectively compared our results in term of AGA identification and delivered “matched therapy” with those we would have obtained using smaller gene panels such as the two US FDA-authorized panels: FoundationOne CDx (coding regions of 309 genes) and MSK-IMPACT (coding regions of 467 genes). Out of the 95 genes concerned by the AGAs we identified, only 12 (13%) are not included in the FoundationOne CDx panel and only 8 (8%) are not included in the MSK-IMPACT panel. In both cases, none of these genes had led to delivery of “matched therapy” in our patients (Additional file 3: Table S8), and the number of patients with AGAs identified with these two commercial panels would have been 392 *versus* 393 with our panel. The HRD score cannot be estimated using the two commercial gene panels, but it was used to deliver “matched therapy” in only five of our 94 treated patients and with clinical benefit in one patient (disease stability and PFS2/PFS1 ratio = 1.91). Thus, our clinical results in the present series would have been very similar using one of the two smaller FDA-authorized panels. Further comparisons are required in the future, integrating not only the single-gene alterations, but also genomic scores such as HRD, TMB, and MSI. This issue regarding the optimal gene panel size is being addressed prospectively in randomized clinical trials, such as ProfiLER-02 that compares a Foundation One Medicine panel (324 genes) and a limited CONTROL panel (87 genes) (NCT03163732). The assessment of highly actionable fusion genes should also be considered. That was not possible with our gene panel. However, fusion genes became more prominent in recent years in epithelial cancers [52] and their potential in precision medicine is well suggested by recent studies. For example, in the MSK-IMPACT initiative [41], 35% of all gene fusions (268 fusions) involved kinase genes and encompassed all or part of the kinase domain, and many known recurrent fusions were found in previously unreported cancer types.

Other roads for improvement concern the relevance of AGAs. Clearly, use of genomic scores such as the frequent HRD status we report, and use of more sophisticated algorithms to better define the functionality and relevance of alteration(s) are required but deserve further ameliorations. Other suggested solutions include the use of a matching score [40], combination of t-NGS with RNA-seq [53], or timely recommendations for individualized treatment with combination therapies [54], notably in patients with multiple AGAs. Of course, such improvements will increase the confidence in precision medicine from patients and physicians and will thus favor inclusion in clinical trials. Other ways to improve the efficacy of precision medicine concern the patients and access to drugs and clinical trials. In our study and other published studies, most reasons for no further therapy or no inclusion in clinical trials of patients with AGA were related to patients’ deterioration. These studies included end-staged metastatic patients previously heavily pretreated—sometimes with targeted therapies before inclusion, often showing non-inclusion criteria for clinical trials, and displaying rapid disease progression sometimes non compatible with the median 2-month time we observed for discussing the molecular results. Such a relative long time is in agreement with that reported in several French precision medicine studies: 40 days in MOSCATO [15], 60 in EXOMA study [16], 63 in the Institut Bergonié’s study [14], and 86 in ProfiLER [17]. Clearly, it remains a limitation that requires improvements in the future. Furthermore, inclusion of patients with various tumor types and pathological types introduce an important source of variability into the analysis that may bias the results, notably because the predictive impact of a molecular alteration depends on the cancer type. In this context, the imbalance of our series regarding the tumor types in favor of breast cancer might make our results not as easily generalizable to all solid cancer types. However, we observed similar efficacy results when analysis was adjusted upon the cancer type (breast cancer *versus* non-breast cancer type). Thus, inclusion of patients earlier in the disease course (lesser risk of clinical deterioration and less complex genomic profile) and with unique cancer type is being tested in trials such as SAFIR02-breast and SAFIR02-lung (NCT02117167, NCT02299999) or MULTISARC (NCT03784014). In parallel, improving the number of and accessibility to clinical trials of matched therapies, alone and in combination, with less restrictive patients’ eligibility criteria [55] and wider selection of participating centers will be crucial since it seems that the highest efficacy of precision medicine is observed in patients treated in large academic centers with broad phase I/II trials portfolio [9, 10, 15, 18, 21, 40].

Finally, we found that 13% of patients tested by germline DNA sequencing displayed PGVs and that more than 85% of concerned genes were involved in DNA repair. This is in agreement with the 12.2% rate reported in a series of 500 metastatic patients [20]. PGV identification in PERMED-01 led to recommend an oncogenetics consultation for 19 patients, of which only five could be carried out because of frequent patient’s death. If this high rate of PGV identification should lead to systematic oncogenetics consultation and germline sequencing in metastatic patients remains to be investigated. However, such rate and the high frequency of altered DNA repair genes suggest that germline sequencing should be associated with somatic sequencing in precision medicine trials for two reasons: therapeutic use of PARP inhibitors or immune checkpoint inhibitors in the cases of HRD or MSI respectively, and help for interpretation of results.

## Conclusions

Such extensive molecular screening trial based on a new tumor biopsy was feasible and allowed identification of AGA in 71% of heavily pretreated metastatic patients and delivery of “matched therapy” on progression in 17%, which improved PFS in 36% of cases. Delivery of “matched therapy” was the sole variable associated with improved PFS2/PFS1 ratio in patients with AGA. We also report for the first time that WES does not provide benefit as compared to t-NGS/aCGH based on a large list of candidate genes. The strengths of our study include prospective design, mandatory new biopsy for analyses, aCGH and NGS profiling using the largest gene panel to date, inclusion of genomic scores as AGAs, and first face-to-face AGA comparison of WES *versus* t-NGS/aCGH data. Limitations include the following: non-randomized nature, profiling of diverse tumor types, imbalance regarding the tumor types in favor of breast cancer, absence of tested bone lesions, median of three prior lines of chemotherapy for advanced disease, testing limited to t-NGS/aCGH, use of different gene panels with time, no detection of fusion genes and other clinically relevant transcriptional alterations [56], use of a relatively little stringent AGA definition, selection of only one method for HRD scoring related to the use of aCGH for profiling, profiling of new biopsied and frozen samples that might limit the applicability of our results to the routine hospital setting where mostly FFPE samples and usually archived samples are being used, choice of therapy (matched *versus* non-matched) by the physician not locked down and preassigned, and matched treatment in phase I studies with doses and schedules imperfect by nature. Clearly, well-designed randomized trials are required to demonstrate the clinical utility of precision medicine: they should enroll patients earlier in the metastatic disease course, use more reliable predictive tools to match patients to the most promising therapies, and offer simpler access to innovative MTT alone and in combination. Evaluation of cost-effectiveness of cancer precision medicine is also required, given the strong deployment of human and financial resources. Today, given the absence of demonstrated clinical benefit and the limited number of both matched therapies and validated gene targets, precision medicine based on genome profiling cannot be used in routine practice and should be reserved to prospective clinical trials not only to show its clinical utility but also to feed both translational and fundamental research.

## Supplementary Information

**Additional file 1.** PERMED-01 protocol. The protocol of PERMED-01 trial.

**Additional file 2: Supplementary methods**. Contains additional methods regarding the trial design, the genome analyses, the molecular precision oncology report and molecular tumor board, and the statistical analyses.

**Additional file 3: Supplementary Tables**. Contains eight Supplementary Tables regarding gene lists, patients’ characteristics, univariate analyses, efficacy results, and pan-cancer HRD rates.

**Additional file 4: Supplementary Figures**. Contains four Supplementary Figures showing the cancer types profiled and further NGA and aCGH results.

## Data Availability

The dataset supporting the conclusions of this article has been deposited in the European Genome-phenome Archive (EGA) repository: accession EGAS00001004554 and https://www.ebi.ac.uk/ega/home for t-NGS data (https://ega-archive.org/studies/EGAS00001004554) [37] and in the ArrayExpress database at EMBL-EBI under the E-MTAB-9998 accession number for array-CGH data (https://www.ebi.ac.uk/arrayexpress/experiments/E-MTAB-9998/) [38]. WES public data was previously available at the EGA repository: accession EGAS00001003290 (https://ega-archive.org/studies/EGAS00001003290) [33].

## Abbreviations

aCGH: Array-comparative genomic hybridization
AGAs: Actionable genetic alterations
BROCA: Breast and ovarian cancer
CI: Confidence interval
CNAs: Copy number alterations
CR: Complete response
DC: Disease control
ECOG: Eastern Cooperative Oncology Group
FDA: Food and Drug Administration
HR: Hazard ratio
HRD: Homologous recombination deficiency
LOH: Loss of heterozygosity
MSI-H: Microsatellite instability high
MTB: Molecular tumor board
MTT: Molecularly targeted therapies
NGS: Next-generation sequencing
OS: Overall survival
PD: Progressive disease
PFS: Progression-free survival
PGVs: Pathogenic germline variants
PR: Partial response
RNA-seq: RNA-sequencing
SD: Stable disease
TMB: Tumor mutational burden
t-NGS: Targeted NGS
WES: Whole-exome sequencing

## References

[1] Lawrence MS, Stojanov P, Polak P, Kryukov GV, Cibulskis K, Sivachenko A, Carter SL, Stewart C, Mermel CH, Roberts SA, Kiezun A, Hammerman PS, McKenna A, Drier Y, Zou L, Ramos AH, Pugh TJ, Stransky N, Helman E, Kim J, Sougnez C, Ambrogio L, Nickerson E, Shefler E, Cortés ML, Auclair D, Saksena G, Voet D, Noble M, DiCara D, Lin P, Lichtenstein L, Heiman DI, Fennell T, Imielinski M, Hernandez B, Hodis E, Baca S, Dulak AM, Lohr J, Landau DA, Wu CJ, Melendez-Zajgla J, Hidalgo-Miranda A, Koren A, McCarroll SA, Mora J, Lee RS, Crompton B, Onofrio R, Parkin M, Winckler W, Ardlie K, Gabriel SB, Roberts CWM, Biegel JA, Stegmaier K, Bass AJ, Garraway LA, Meyerson M, Golub TR, Gordenin DA, Sunyaev S, Lander ES, Getz G (2013). Mutational heterogeneity in cancer and the search for new cancer-associated genes. Nature..

[2] Hyman DM, Puzanov I, Subbiah V, Faris JE, Chau I, Blay JY, Wolf J, Raje NS, Diamond EL, Hollebecque A, Gervais R, Elez-Fernandez ME, Italiano A, Hofheinz RD, Hidalgo M, Chan E, Schuler M, Lasserre SF, Makrutzki M, Sirzen F, Veronese ML, Tabernero J, Baselga J (2015). Vemurafenib in multiple nonmelanoma cancers with BRAF V600 mutations. N Engl J Med..

[3] Iyer G, Hanrahan AJ, Milowsky MI, Al-Ahmadie H, Scott SN, Janakiraman M, Pirun M, Sander C, Socci ND, Ostrovnaya I (2012). Genome sequencing identifies a basis for everolimus sensitivity. Science..

[4] Chanez B, Chaffanet M, Adelaide J, Thomassin J, Garnier S, Carbuccia N, Guille A, Charrier N, Brenot-Rossi I, Pignot G (2018). De novo metastatic small cell carcinoma of the prostate with BRCA2 mutation: report of a successful precision medicine management with PARP inhibitors. JCO Precis Oncol..

[5] Sabatier R, Lopez M, Guille A, Billon E, Carbuccia N, Garnier S, Adelaide J, Extra JM, Capiello MA, Charafe-Jauffret E (2019). High response to cetuximab for a patient with EGFR-amplified heavily pretreated metastatic triple-negative breast cancer. JCO Precis Oncol..

[6] Tsimberidou AM, Fountzilas E, Nikanjam M, Kurzrock R (2020). Review of precision cancer medicine: evolution of the treatment paradigm. Cancer Treat Rev..

[7] Yates LR, Seoane J, Le Tourneau C, Siu LL, Marais R, Michiels S, Soria JC, Campbell P, Normanno N, Scarpa A (2018). The European Society for Medical Oncology (ESMO) Precision Medicine Glossary. Ann Oncol..

[8] Von Hoff DD, Stephenson JJ, Rosen P, Loesch DM, Borad MJ, Anthony S, Jameson G, Brown S, Cantafio N, Richards DA (2010). Pilot study using molecular profiling of patients’ tumors to find potential targets and select treatments for their refractory cancers. J Clin Oncol..

[9] Tsimberidou AM, Iskander NG, Hong DS, Wheler JJ, Falchook GS, Fu S, Piha-Paul S, Naing A, Janku F, Luthra R, Ye Y, Wen S, Berry D, Kurzrock R (2012). Personalized medicine in a phase I clinical trials program: the MD Anderson Cancer Center initiative. Clin Cancer Res..

[10] Tsimberidou AM, Wen S, Hong DS, Wheler JJ, Falchook GS, Fu S, Piha-Paul S, Naing A, Janku F, Aldape K, Ye Y, Kurzrock R, Berry D (2014). Personalized medicine for patients with advanced cancer in the phase I program at MD Anderson: validation and landmark analyses. Clin Cancer Res..

[11] Kim ES, Herbst RS, Wistuba II, Lee JJ, Blumenschein GR, Tsao A, Stewart DJ, Hicks ME, Erasmus J, Gupta S, Alden CM, Liu S, Tang X, Khuri FR, Tran HT, Johnson BE, Heymach JV, Mao L, Fossella F, Kies MS, Papadimitrakopoulou V, Davis SE, Lippman SM, Hong WK (2011). The BATTLE trial: personalizing therapy for lung cancer. Cancer Discov..

[12] Kris MG, Johnson BE, Berry LD, Kwiatkowski DJ, Iafrate AJ, Wistuba II, Varella-Garcia M, Franklin WA, Aronson SL, Su PF, Shyr Y, Camidge DR, Sequist LV, Glisson BS, Khuri FR, Garon EB, Pao W, Rudin C, Schiller J, Haura EB, Socinski M, Shirai K, Chen H, Giaccone G, Ladanyi M, Kugler K, Minna JD, Bunn PA (2014). Using multiplexed assays of oncogenic drivers in lung cancers to select targeted drugs. JAMA..

[13] Andre F, Bachelot T, Commo F, Campone M, Arnedos M, Dieras V, Lacroix-Triki M, Lacroix L, Cohen P, Gentien D (2014). Comparative genomic hybridisation array and DNA sequencing to direct treatment of metastatic breast cancer: a multicentre, prospective trial (SAFIR01/UNICANCER). Lancet Oncol..

[14] Cousin S, Grellety T, Toulmonde M, Auzanneau C, Khalifa E, Laizet Y, Tran K, Le Moulec S, Floquet A, Garbay D (2017). Clinical impact of extensive molecular profiling in advanced cancer patients. J Hematol Oncol..

[15] Massard C, Michiels S, Ferte C, Le Deley MC, Lacroix L, Hollebecque A, Verlingue L, Ileana E, Rosellini S, Ammari S (2017). High-throughput genomics and clinical outcome in hard-to-treat advanced cancers: results of the MOSCATO 01 trial. Cancer Discov..

[16] Reda M, Richard C, Bertaut A, Niogret J, Collot T, Fumet JD, Blanc J, Truntzer C, Desmoulins I, Ladoire S (2020). Implementation and use of whole exome sequencing for metastatic solid cancer. EBioMedicine..

[17] Tredan O, Wang Q, Pissaloux D, Cassier P, de la Fouchardiere A, Fayette J, Desseigne F, Ray-Coquard I, de la Fouchardiere C, Frappaz D (2019). Molecular screening program to select molecular-based recommended therapies for metastatic cancer patients: analysis from the ProfiLER trial. Ann Oncol..

[18] Tsimberidou AM, Hong DS, Ye Y, Cartwright C, Wheler JJ, Falchook GS, et al. Initiative for Molecular Profiling and Advanced Cancer Therapy (IMPACT): an MD Anderson Precision Medicine Study. JCO Precis Oncol. 2017;PO.17.00002. 10.1200/PO.17.00002. Epub 2017 Sep 8. 10.1200/PO.17.00002 PMC5659750 29082359

[19] Pishvaian MJ, Bender RJ, Halverson D, Rahib L, Hendifar AE, Mikhail S, Chung V, Picozzi VJ, Sohal D, Blais EM, Mason K, Lyons EE, Matrisian LM, Brody JR, Madhavan S, Petricoin EF (2018). Molecular profiling of patients with pancreatic cancer: initial results from the know your tumor initiative. Clin Cancer Res..

[20] Robinson DR, Wu YM, Lonigro RJ, Vats P, Cobain E, Everett J, Cao X, Rabban E, Kumar-Sinha C, Raymond V, Schuetze S, Alva A, Siddiqui J, Chugh R, Worden F, Zalupski MM, Innis J, Mody RJ, Tomlins SA, Lucas D, Baker LH, Ramnath N, Schott AF, Hayes DF, Vijai J, Offit K, Stoffel EM, Roberts JS, Smith DC, Kunju LP, Talpaz M, Cieślik M, Chinnaiyan AM (2017). Integrative clinical genomics of metastatic cancer. Nature..

[21] Schwaederle M, Parker BA, Schwab RB, Daniels GA, Piccioni DE, Kesari S, Helsten TL, Bazhenova LA, Romero J, Fanta PT, Lippman SM, Kurzrock R (2016). Precision Oncology: The UC San Diego Moores Cancer Center PREDICT Experience. Mol Cancer Ther..

[22] Scott RJ, Sobol HH (1999). Prognostic implications of cancer susceptibility genes: any news?. Recent Results Cancer Res..

[23] Stockley TL, Oza AM, Berman HK, Leighl NB, Knox JJ, Shepherd FA, Chen EX, Krzyzanowska MK, Dhani N, Joshua AM, Tsao MS, Serra S, Clarke B, Roehrl MH, Zhang T, Sukhai MA, Califaretti N, Trinkaus M, Shaw P, van der Kwast T, Wang L, Virtanen C, Kim RH, Razak ARA, Hansen AR, Yu C, Pugh TJ, Kamel-Reid S, Siu LL, Bedard PL (2016). Molecular profiling of advanced solid tumors and patient outcomes with genotype-matched clinical trials: the Princess Margaret IMPACT/COMPACT trial. Genome Med..

[24] Remon J, Dienstmann R (2018). Precision oncology: separating the wheat from the chaff. ESMO Open..

[25] Swanton C, Soria JC, Bardelli A, Biankin A, Caldas C, Chandarlapaty S, de Koning L, Dive C, Feunteun J, Leung SY, Marais R, Mardis ER, McGranahan N, Middleton G, Quezada SA, Rodón J, Rosenfeld N, Sotiriou C, André F (2016). Consensus on precision medicine for metastatic cancers: a report from the MAP conference. Ann Oncol..

[26] McGranahan N, Favero F, de Bruin EC, Birkbak NJ, Szallasi Z, Swanton C (2015). Clonal status of actionable driver events and the timing of mutational processes in cancer evolution. Sci Transl Med.

[27] Bertucci F, Ng CKY, Patsouris A, Droin N, Piscuoglio S, Carbuccia N, Soria JC, Dien AT, Adnani Y, Kamal M, Garnier S, Meurice G, Jimenez M, Dogan S, Verret B, Chaffanet M, Bachelot T, Campone M, Lefeuvre C, Bonnefoi H, Dalenc F, Jacquet A, de Filippo MR, Babbar N, Birnbaum D, Filleron T, le Tourneau C, André F (2019). Genomic characterization of metastatic breast cancers. Nature..

[28] Tyran M, Carbuccia N, Garnier S, Guille A, Adelaide J, Finetti P, Toulzian J, Viens P, Tallet A, Goncalves A, et al. A comparison of DNA mutation and copy number profiles of primary breast cancers and paired brain metastases for identifying clinically relevant genetic alterations in brain metastases. Cancers (Basel). 2019;11(5):665. 10.3390/cancers11050665. 10.3390/cancers11050665 PMC6562582 31086113

[29] Abkevich V, Timms KM, Hennessy BT, Potter J, Carey MS, Meyer LA, Smith-McCune K, Broaddus R, Lu KH, Chen J, Tran TV, Williams D, Iliev D, Jammulapati S, FitzGerald LM, Krivak T, DeLoia JA, Gutin A, Mills GB, Lanchbury JS (2012). Patterns of genomic loss of heterozygosity predict homologous recombination repair defects in epithelial ovarian cancer. Br J Cancer..

[30] Bertucci F, Finetti P, Guille A, Adelaide J, Garnier S, Carbuccia N, Monneur A, Charafe-Jauffret E, Goncalves A, Viens P (2016). Comparative genomic analysis of primary tumors and metastases in breast cancer. Oncotarget..

[31] Helleman J, Jansen MP, Span PN, van Staveren IL, Massuger LF, Meijer-van Gelder ME, Sweep FC, Ewing PC, van der Burg ME, Stoter G (2006). Molecular profiling of platinum resistant ovarian cancer. Int J Cancer..

[32] Niu B, Ye K, Zhang Q, Lu C, Xie M, McLellan MD, Wendl MC, Ding L (2014). MSIsensor: microsatellite instability detection using paired tumor-normal sequence data. Bioinformatics..

[33] Bertucci F, Ng CKY, Patsouris A, Droin N, Piscuoglio N, Carbuccia N, Soria JC, Tran Dien A, Adnani Y, Kamal M, et al. Whole-exome sequencing data. EGAS00001003290. European Genome-phenome Archive database. https://ega-archive.org/datasets/EGAS00001003290. (2019). Accessed 16 Aug 2020.

[34] Marquard AM, Eklund AC, Joshi T, Krzystanek M, Favero F, Wang ZC, Richardson AL, Silver DP, Szallasi Z, Birkbak NJ (2015). Pan-cancer analysis of genomic scar signatures associated with homologous recombination deficiency suggests novel indications for existing cancer drugs. Biomark Res..

[35] Telli ML, Timms KM, Reid J, Hennessy B, Mills GB, Jensen KC, Szallasi Z, Barry WT, Winer EP, Tung NM, Isakoff SJ, Ryan PD, Greene-Colozzi A, Gutin A, Sangale Z, Iliev D, Neff C, Abkevich V, Jones JT, Lanchbury JS, Hartman AR, Garber JE, Ford JM, Silver DP, Richardson AL (2016). Homologous recombination deficiency (HRD) score predicts response to platinum-containing neoadjuvant chemotherapy in patients with triple-negative breast cancer. Clin Cancer Res..

[36] Chakravarty D, Gao J, Phillips SM, Kundra R, Zhang H, Wang J, et al. OncoKB: a precision oncology knowledge base. JCO Precis Oncol. 2017;PO.17.00011. 10.1200/PO.17.00011. Epub 2017 May 16. 10.1200/PO.17.00011 PMC5586540 28890946

[37] Bertucci F, Gonçalves A, Guille A, Adelaide J, Garnier S, Carbuccia N, Billon E, Finetti P, et al. Targeted NGS data. EGAS00001004554. EuropeanGenome-phenome Archive database. https://ega-archive.org/studies/EGAS00001004554. Accessed 15 July 2020.

[38] Bertucci F, Gonçalves A, Guille A, Adelaide J, Garnier S, Carbuccia N, Billon E, Finetti P, et al. Array-CGH data. E-MTAB-9998. ArrayExpress database. https://www.ebi.ac.uk/arrayexpress/experiments/E-MTAB-9998. (2021). Accessed 6 Jan 2021.

[39] Plon SE, Eccles DM, Easton D, Foulkes WD, Genuardi M, Greenblatt MS, Hogervorst FB, Hoogerbrugge N, Spurdle AB, Tavtigian SV, Group IUGVW (2008). Sequence variant classification and reporting: recommendations for improving the interpretation of cancer susceptibility genetic test results. Hum Mutat..

[40] Wheler JJ, Janku F, Naing A, Li Y, Stephen B, Zinner R, Subbiah V, Fu S, Karp D, Falchook GS, Tsimberidou AM, Piha-Paul S, Anderson R, Ke D, Miller V, Yelensky R, Lee JJ, Hong DS, Kurzrock R (2016). Cancer therapy directed by comprehensive genomic profiling: a single center study. Cancer Res..

[41] Zehir A, Benayed R, Shah RH, Syed A, Middha S, Kim HR, Srinivasan P, Gao J, Chakravarty D, Devlin SM, Hellmann MD, Barron DA, Schram AM, Hameed M, Dogan S, Ross DS, Hechtman JF, DeLair DF, Yao JJ, Mandelker DL, Cheng DT, Chandramohan R, Mohanty AS, Ptashkin RN, Jayakumaran G, Prasad M, Syed MH, Rema AB, Liu ZY, Nafa K, Borsu L, Sadowska J, Casanova J, Bacares R, Kiecka IJ, Razumova A, Son JB, Stewart L, Baldi T, Mullaney KA, al-Ahmadie H, Vakiani E, Abeshouse AA, Penson AV, Jonsson P, Camacho N, Chang MT, Won HH, Gross BE, Kundra R, Heins ZJ, Chen HW, Phillips S, Zhang H, Wang J, Ochoa A, Wills J, Eubank M, Thomas SB, Gardos SM, Reales DN, Galle J, Durany R, Cambria R, Abida W, Cercek A, Feldman DR, Gounder MM, Hakimi AA, Harding JJ, Iyer G, Janjigian YY, Jordan EJ, Kelly CM, Lowery MA, Morris LGT, Omuro AM, Raj N, Razavi P, Shoushtari AN, Shukla N, Soumerai TE, Varghese AM, Yaeger R, Coleman J, Bochner B, Riely GJ, Saltz LB, Scher HI, Sabbatini PJ, Robson ME, Klimstra DS, Taylor BS, Baselga J, Schultz N, Hyman DM, Arcila ME, Solit DB, Ladanyi M, Berger MF (2017). Mutational landscape of metastatic cancer revealed from prospective clinical sequencing of 10,000 patients. Nat Med..

[42] Mosele F, Remon J, Mateo J, Westphalen CB, Barlesi F, Lolkema MP, Normanno N, Scarpa A, Robson M, Meric-Bernstam F, Wagle N, Stenzinger A, Bonastre J, Bayle A, Michiels S, Bièche I, Rouleau E, Jezdic S, Douillard JY, Reis-Filho JS, Dienstmann R, André F (2020). Recommendations for the use of next-generation sequencing (NGS) for patients with metastatic cancers: a report from the ESMO Precision Medicine Working Group. Ann Oncol..

[43] Diossy M, Reiniger L, Sztupinszki Z, Krzystanek M, Timms KM, Neff C, Solimeno C, Pruss D, Eklund AC, Toth E (2018). Breast cancer brain metastases show increased levels of genomic aberration-based homologous recombination deficiency scores relative to their corresponding primary tumors. Ann Oncol..

[44] Sun J, Wang C, Zhang Y, Xu L, Fang W, Zhu Y, Zheng Y, Chen X, Xie X, Hu X, Hu W, Zheng J, Li P, Yu J, Mei Z, Cai X, Wang B, Hu Z, Shu Y, Shen H, Gu Y (2019). Genomic signatures reveal DNA damage response deficiency in colorectal cancer brain metastases. Nat Commun..

[45] Le Tourneau C, Delord JP, Goncalves A, Gavoille C, Dubot C, Isambert N, Campone M, Tredan O, Massiani MA, Mauborgne C (2015). Molecularly targeted therapy based on tumour molecular profiling versus conventional therapy for advanced cancer (SHIVA): a multicentre, open-label, proof-of-concept, randomised, controlled phase 2 trial. Lancet Oncol..

[46] Pishvaian MJ, Blais EM, Brody JR, Lyons E, DeArbeloa P, Hendifar A, Mikhail S, Chung V, Sahai V, Sohal DPS, Bellakbira S, Thach D, Rahib L, Madhavan S, Matrisian LM, Petricoin EF (2020). Overall survival in patients with pancreatic cancer receiving matched therapies following molecular profiling: a retrospective analysis of the Know Your Tumor registry trial. Lancet Oncol..

[47] Aisner DL, Sholl LM, Berry LD, Rossi MR, Chen H, Fujimoto J, Moreira AL, Ramalingam SS, Villaruz LC, Otterson GA, Haura E, Politi K, Glisson B, Cetnar J, Garon EB, Schiller J, Waqar SN, Sequist LV, Brahmer J, Shyr Y, Kugler K, Wistuba II, Johnson BE, Minna JD, Kris MG, Bunn PA, Kwiatkowski DJ, LCMC2 investigators (2018). The impact of smoking and TP53 mutations in lung adenocarcinoma patients with targetable mutations-the Lung Cancer Mutation Consortium (LCMC2). Clin Cancer Res..

[48] Papadimitrakopoulou V, Lee JJ, Wistuba II, Tsao AS, Fossella FV, Kalhor N, Gupta S, Byers LA, Izzo JG, Gettinger SN, Goldberg SB, Tang X, Miller VA, Skoulidis F, Gibbons DL, Shen L, Wei C, Diao L, Peng SA, Wang J, Tam AL, Coombes KR, Koo JS, Mauro DJ, Rubin EH, Heymach JV, Hong WK, Herbst RS (2016). The BATTLE-2 study: a biomarker-integrated targeted therapy study in previously treated patients with advanced non-small-cell lung cancer. J Clin Oncol..

[49] Lee J, Kim ST, Kim K, Lee H, Kozarewa I, Mortimer PGS, Odegaard JI, Harrington EA, Lee J, Lee T, Oh SY, Kang JH, Kim JH, Kim Y, Ji JH, Kim YS, Lee KE, Kim J, Sohn TS, An JY, Choi MG, Lee JH, Bae JM, Kim S, Kim JJ, Min YW, Min BH, Kim NKD, Luke S, Kim YH, Hong JY, Park SH, Park JO, Park YS, Lim HY, Talasaz A, Hollingsworth SJ, Kim KM, Kang WK (2019). Tumor genomic profiling guides patients with metastatic gastric cancer to targeted treatment: the VIKTORY Umbrella Trial. Cancer Discov..

[50] Schwaederle M, Zhao M, Lee JJ, Lazar V, Leyland-Jones B, Schilsky RL, Mendelsohn J, Kurzrock R (2016). Association of biomarker-based treatment strategies with response rates and progression-free survival in refractory malignant neoplasms: a meta-analysis. JAMA Oncol..

[51] Schwaederle M, Zhao M, Lee JJ, Eggermont AM, Schilsky RL, Mendelsohn J, Lazar V, Kurzrock R (2015). Impact of precision medicine in diverse cancers: a meta-analysis of phase II clinical trials. J Clin Oncol..

[52] Kumar-Sinha C, Kalyana-Sundaram S, Chinnaiyan AM (2015). Landscape of gene fusions in epithelial cancers: seq and ye shall find. Genome Med..

[53] Rodon J, Soria JC, Berger R, Miller WH, Rubin E, Kugel A, Tsimberidou A, Saintigny P, Ackerstein A, Brana I (2019). Genomic and transcriptomic profiling expands precision cancer medicine: the WINTHER trial. Nat Med..

[54] Sicklick JK, Kato S, Okamura R, Schwaederle M, Hahn ME, Williams CB, De P, Krie A, Piccioni DE, Miller VA (2019). Molecular profiling of cancer patients enables personalized combination therapy: the I-PREDICT study. Nat Med..

[55] Kim ES, Bruinooge SS, Roberts S, Ison G, Lin NU, Gore L, Uldrick TS, Lichtman SM, Roach N, Beaver JA, Sridhara R, Hesketh PJ, Denicoff AM, Garrett-Mayer E, Rubin E, Multani P, Prowell TM, Schenkel C, Kozak M, Allen J, Sigal E, Schilsky RL (2017). Broadening eligibility criteria to make clinical trials more representative: American Society of Clinical Oncology and Friends of Cancer Research Joint Research Statement. J Clin Oncol..

[56] Tsimberidou AM, Fountzilas E, Bleris L, Kurzrock R. Transcriptomics and solid tumors: The next frontier in precision cancer medicine. Semin Cancer Biol. 2020. 10.1016/j.semcancer.2020.09.007. 10.1016/j.semcancer.2020.09.007 PMC11927324 32950605

