# Supplementary material for: Prospective high-throughput genome profiling of advanced cancers: results of the PERMED-01 clinical trial
Source: Genome Med. 2021 May 18;13:87. doi: 10.1186/s13073-021-00897-9 (PMC8132379; doi:10.1186/s13073-021-00897-9)
Supplement: Supplementary file 1 — Additional file 1. PERMED-01 protocol. The protocol of PERMED-01 trial. [file 13073_2021_897_MOESM1_ESM.pdf]

INSTITUT PAOLI-CALMETTES

CENTRE RÉGIONAL DE LUTTE CONTRE LE CANCER
PROVENCE-ALPES-CÔTE D'AZUR

PROTOCOL

IDENTIFYING MOLECULAR ALTERATIONS TO GUIDE INDIVIDUALIZED TREATMENT IN ADVANCED SOLID TUMORS: PERMED01 TRIAL

PERMED01-IPC 2014-003
Study Registration No. ID-RCB: 2014-A00966-41

Modification No. 4 – Version No. 5.0 – 22/05/2018 approved by the Ethics Committee and ANSM

Principal Investigator

Pr. François BERTUCCI
Paoli-Calmettes Institute
Department of Medical Oncology
232 Boulevard Ste Marguerite – BP 156
13273 MARSEILLE CEDEX 9
Tel: +33 04 91 22 35 37 - Fax: +33 04 91 22 36 70
Email: bertuccif@ipc.unicancer.fr

Sponsor

Paoli-Calmettes Institute
232 Boulevard Ste Marguerite – BP 156
13273 MARSEILLE CEDEX 9
Tel: +33 04 91 22 37 78 - Fax: +33 04 91 22 36 01
Email: drci.up@ipc.unicancer.fr

Any partial or total reproduction of this document is prohibited without permission of the IPC.

☐ Drug
☐ Blood Products, Organs, Tissues, Cell Therapy Preparations
☐ Medical Device
☒ Outside of article L.5311-1 of the public health code

PROTOCOL APPROVAL AND SIGNATORIES

STUDY OF MOLECULAR ALTERATIONS FOR TARGETED THERAPIES IN ADVANCED SOLID TUMORS - PERMED01-IPC 2014-003

COMPETENT AUTHORITY	*Agence Nationale de Sécurité du Médicament et des Produits de Santé* (ANSM, National Agency for Medicines and Health Products Safety)	Authorization Date: 21/07/2014
		Ref. ANSM: 140823B-12
ETHICS COMMITTEE	Name: *Comité de Protection des Personnes Sud Méditerranée I* (South Mediterranean I Ethics Committee)	Notice Date: 09/07/2014
		Ref. 1456

DRAFTING COMMITTEE	François BERTUCCI, Anthony GONÇALVES, Jihane PAKRADOUNI/DEMEESTÈRE

NAMES AND TITLES OF THOSE RESPONSIBLE FOR THE STUDY	CONTACT INFORMATION	DATE (dd-mm-yy)	SIGNATURE
Principal investigator/coordinator<br>**Pr. François BERTUCCI**	Département d'Oncologie Médicale<br>*Institut Paoli-Calmettes*<br>232 bd Sainte Marguerite – BP 156<br>13273 Marseille Cedex 9<br>Tel:+33 04 91 22 35 37<br>Email: bertuccif@ipc.unicancer.fr		
Department Director<br>**Dr. Dominique GENRE**	*Département de la Recherche Clinique et de l'Innovation*<br>Paoli-Calmettes Institute<br>232 bd Sainte Marguerite – BP 156<br>13273 Marseille Cedex 9<br>Tel: +33 04 91 22 37 78<br>Fax: 04 91 22 36 01<br>Email: drci.up@ipc.unicancer.fr		
Project Head<br>**Dr. Jihane PAKRADOUNI/DEMEESTERE**			
Biostatistician<br>**Christophe ZEMMOUR**			
Data manager<br>**Virginie MARTIN**			

The research will be conducted in accordance with the protocol, the standard of good clinical practice, and the current legislative and regulatory provisions.

TABLE OF CONTENTS

I - RATIONALE 4
1.1 Cancer biology and targeted therapies 4
1.2 High-throughput molecular characterization of tumors 4
1.3 Personalized medicine in advanced stages 4
1.4 The PERMED01 project 5
II – OBJECTIVES 5
2.1 Primary objective 5
2.2 Secondary objectives 5
III – ELIGIBILITY CRITERIA 6
3.1 Inclusion criteria 6
3.2 Exclusion criteria 6
3.3 Patient registration 6
IV – STUDY DESIGN 7
4.1 Research methodology 7
4.2 – Biological sampling at inclusion (cf Appendix 2) 7
4.2.1 - Tumor samples (locally advanced tumor or metastasis): biopsies or surgical specimens.. 7
4.2.2 - Blood samples 8
4.3 – Clinical data 8
4.4 – Molecular analyses 9
4.4.1 - Testing for copy number alterations: CGH-array 9
4.4.2 - Testing for mutations: targeted sequencing (NGS) 9
4.4.3 - Validation of protein expression of relevant targets: IHC 9
4.4.4 - Bioinformatics analysis of molecular data 9
4.4.5 - Complementary molecular analyses. 9
4.5 – Patient care 10
V – EVALUATION OF SERIOUS ADVERSE EVENTS 10
5.1 Definition 10
5.2 Definition of expected serious adverse events (E-SAE) 11
5.3 Definition of a suspected unexpected adverse reaction (SUSAR) 11
5.4 Course of action in the case of a serious adverse event 11
VI – STATISTICS 12
6.1 Description of planned statistical methods 12
6.2 Expected number of research participants to be included 12
VII – RIGHT OF ACCESS TO DATA AND SOURCE DOCUMENTS 13
VIII – QUALITY CONTROL AND ASSURANCE 13
IX – ETHICAL CONSIDERATIONS 13
9.1 Regulations 13
9.2 Ethics committee 14
9.3 Declaration to the competent authorities 14
9.4 Patient informed consent 14
9.5 Investigators' responsibilities 14
9.6 Sponsor's responsibilities 15
X – DATA PROCESSING AND RETENTION OF DOCUMENTS AND DATA RELATING TO RESEARCH 15
XI – CONFIDENTIALITY AND PROPERTIES OF RESEARCH DATA 15
XII – INSURANCE 16
XIII – RULES RELATING TO PUBLICATION 16
XIV - BIBLIOGRAPHY 17
XV – APPENDICES 20
Appendix 1: ECOG (Eastern Cooperative Oncology Group) performance scale 21
Appendix 2: Investigations summary 22
Appendix 3: Validation of preclinical models of therapeutic response in locally advanced or metastatic breast tumors 23
Appendix 4: Study of circulating tumor DNA 26
Appendix 5: Study of circulating tumor cells 27

I - RATIONALE

1.1. Cancer biology and targeted therapies

The management of cancer patients, historically based on histological/clinical diagnostic and prognostic parameters (site, histological type, etc.) and on cytotoxic treatments (chemotherapy, radiotherapy) targeting both cancerous and healthy cells, has made significant progress over the past two decades thanks to increased knowledge of tumor biology. For example, in breast cancer, the analysis of hormone receptors (expression) and the HER2 tyrosine kinase receptor (expression and/or amplification) is now routinely carried out for not only prognosis but also the use of hormone therapy and targeted anti-HER2 therapies (1). Targeted therapies are drugs that can be injected or administered orally that target the signaling pathways that control tumor progression. The toxicity is generally limited. Efficacy is demonstrated in terms of objective response, progression-free survival, and sometimes overall survival, and is all the more important when the targeted protein represents a major and early molecular alteration in the course of the disease (e.g. imatinib that inhibits the mutated Kit receptor in gastrointestinal stromal tumors). These drugs work when the target is present in the disease. This “one target - one drug” concept has been adapted to numerous cancers both in routine clinical practice with around thirty different drugs and in clinical research with several hundred drugs in development.

1.2. High-throughput molecular characterization of tumors

At the molecular level, cancer is characterized by the accumulation and combination of multiple genetic and epigenetic abnormalities responsible for tumor origin, genomic instability, and the acquisition of an invasive and resistant phenotype that results in metastasis and death. For the past fifteen years, high-throughput molecular analyses ("omics") have revolutionized research by making it possible to develop a pan-genomic molecular portrait of a sample (2). The analyses revealed the extraordinary heterogeneity of the tumors and partly explained their histological and clinical heterogeneity. Thus, "organ-specific cancer" is actually comprised of a large number of molecularly distinct diseases that merit individual treatments. Conversely, the cancers of different organs can have identical molecular alterations. This differentiation of cancers and the expansion of the catalog of alterations go hand in hand with the ever-increasing development of innovative targeted therapies. Recently, high-throughput sequencing has revealed a large number of “candidate” mutations (*drivers*) that, while admittedly occurring infrequently (< 5%), are potentially "targetable" by drugs already on the market or in development for the given cancer, other cancers, or other non-cancerous diseases.

1.3. Personalized medicine for advanced cancer stages

In this dynamic context of improved molecular characterization of cancers, development of new targeted therapies, and advances in terms of molecular typing, the therapeutic management must increasingly integrate the specific molecular characteristics of individual tumors. According to the American Food and Drug Administration (FDA), "personalized medicine" aims to obtain "the best medical results by selecting therapeutics according to patient’s genomic profile or other patient’s characteristics such as proteins, either circulating or at the cell surface”. The idea being to deliver treatments based on the molecular typing of the tumor and/or metastasis, regardless of location and histological type, and is currently being applied in research and development. The importance of this approach was demonstrated for the first time by D. Von Hoff's team (3), who used IHC and DNA microarrays to analyze tumor samples from 86 patients with refractory metastatic cancer in order to measure the expression of 61 genes/proteins that were targeted by existing therapies. A potential therapeutic target was detected in 84 patients, 66 of whom then

received the corresponding treatment. For 18 of these, progression-free survival with their treatments lasted more than 1.3 times the length of progression-free survival with previous treatment. The same concept was tested in metastatic breast cancer in the French SAFIR01 trial with promising results. Their analysis of metastatic samples included the search for amplifications in the pan-genome CGH-array and mutations in mTOR pathway (*PI3K*, *PTEN*, *AKT*) genes to see if molecular targets were present that allowed for the referral of the patient to an early trial of targeted therapies. A workable genomic profile was obtained in 64% of patients, 69% of whom had a molecular abnormality of interest for which a targeted drug existed. A targeted personalized treatment was delivered to 43/423 patients with regression or stabilization of the disease of at least 4 months in 13 cases (4). Additional prospective studies are currently underway in France that are testing this concept in patients with all types of metastatic solid tumors: the MOSCATO trial (MOlecular Screening for CAncer Treatment Optimization), sponsored by the Gustave-Roussy Institute; the MOST (My Own Specific Treatment) trial, sponsored by the Léon Bérard Center; and the SHIVA trial, sponsored by the Curie Institute (5). The SHIVA trial plans to evaluate this strategy in a randomized setting for the first time, comparing it to a conventional approach in patients with a molecular abnormality that is potentially targetable by known therapies (imatinib, everolimus, vemurafenib, sorafenib, erlotinib, lapatinib + trastuzumab, dasatinib, tamoxifen, or letrozole). Personalized medicine programs are also underway in several countries: the MD Anderson Cancer Center (6), Dr. Chinayan's team in the USA (7), and Toronto in Canada (8). In these studies, typing is performed on a metastatic sample rather than the original tumor due to the appearance of new molecular alterations and resistant clones during tumor progression that may alter therapeutic indications.

1.4. The PERMED01 project

In this context, the Paoli-Calmettes Institute (IPC) plans to implement the PERMED01 project; a prospective monocentric cohort study collecting clinically annotated tissue samples from patients with advanced solid tumors in order to perform a molecular screening (portrait) of the disease for therapeutic purposes. The IPC has all the necessary knowledge, expertise, and technological tools, particularly in Genomics (9-23), to properly type tumor samples. Our institute has also acquired experience in clinical genomics-based research via three prospective clinical trials, the SA02 trial based on transcriptome analysis and, more recently, the SAFIR01 and Shiva trials. This molecular portrait will complement the research of classical biomarkers (IHC, FISH, sequencing) that has been carried out for years, in addition to providing real-time information on a variety of molecular alterations of interest that could help guide patient treatment.

II – OBJECTIVES

2.1 Primary objective

Define the number of patients whose molecular alteration(s), identified in samples in the context of this study, could lead to the delivery of "targeted" therapy.

2.2 Secondary objectives

- Define the molecular alterations of advanced malignant solid tumors.
- Compare the molecular portraits of metastatic samples with those of the primary tumor if available (heterogeneity, clonal expansion) and normal cells (somatic or constitutional mutations) from the same patient.
- Determine, for the most frequent diseases (such as breast cancer; lung cancer; gynecological cancers, such as cervical, endometrial, ovarian, and fallopian tube; urological cancer, such as prostate, kidney, and bladder; and digestive cancers, such

as colon, pancreatic, and liver), the relationship between these molecular alterations and the histological and clinical disease characteristics, including progression-free survival and overall survival.
- Perform pan-genomic molecular analysis on metastatic samples with full exome sequencing and transcriptome analysis (expression, fusion transcripts: DNA microarray, RNA-seq).
- Perform an analysis of circulating tumor DNA.
- In the case of breast cancer, validate preclinical models of therapeutic tumor response (xenografts, short-term culture, and organoids).
- In cases of breast and digestive cancer, validate the isolation and characterization of circulating tumor cells.

III – ELIGIBILITY CRITERIA

The project is based on the collection and analysis of biological tumor and blood samples for any patient treated at our Institute for advanced malignant solid tumors (metastatic or locally advanced) progressive during at least one line of prior systemic therapy. All cancer types will be included whether they are common or rare cancers for which treatment options are even more limited at an advanced stage.

3.1 Inclusion criteria

- Age ≥ 18 ans.
- Histological cancer diagnosis.
- Locally advanced or metastatic stage, with tumor lesion accessible for biopsy.
- ECOG PS ≤ 2 (Appendix 1).
- Affiliated with or benefiting from social security.
- Signed informed consent form for participation in the study.

3.2 Exclusion criteria

- Person in an emergency situation, an adult who is neither a ward nor a dependent and is not under any type of guardianship or is unable to express consent.
- Progressive brain metastases.
- Brain or bone metastases as the only biopsy site.
- Pregnant or breastfeeding women.

3.3 Patient registration

Patients after having been informed and accepting to participate in the study shall sign an informed consent form. Eligible patients are registered by completely filling out the inclusion form at:

https://www.canceropole-pacacoonline.com/crfonline

Department of Clinical Research and Development (DCRD)
232, Boulevard Ste Marguerite – BP 156
13273 MARSEILLE CEDEX 9
Tel: +33 04 91 22 37 78 - Fax: +33 04 91 22 36 01
Email: drci.up@ipc.unicancer.fr

An inclusion number will be sent via email to the DCRD secretariat, the project head, the clinical research technician, the clinical research associate, the investigator, the biological resources center, and to the pathologist upon confirmation of inclusion.

IV – STUDY DESIGN

4.1 Research methodology

Prospective, open-label, single-center trial.

4.2 – Biological sampling at inclusion (cf Appendix 2)

4.2.1 - Tumor samples (locally advanced tumor or metastasis): biopsies or surgical specimens

The sampling sites will be determined by the investigative team based on its accessibility and volume. Bone and brain metastases will not be sampled. At least 3 tumor fragments will be taken for each patient and sample site selected.

These fragments will be obtained:

- either via biopsy performed with radiological or endoscopic monitoring in the Radiology Department or the Medical-Surgical Exploration Unit (*Unité d'Explorations Médico-Chirurgicales*), respectively. The sample may be taken, depending on the location of the site selected:

    - by percutaneous image-guided biopsy (ultrasound or scanner), mainly using 14, 16, or 18-gauge needles via a manual, semi-automatic, or automatic mechanism. The radiologist will select the most suitable technique and type of needle for each sample.

    - by biopsy at the end of an endoscopic ultrasound examination, mainly using 19 or 22 gauge needles that are 8 cm long. Biopsies are obtained by aspiration with a 20 ml syringe with a back-and-forth movement in the tumor; 1 to 3 passes are necessary to obtain a microbiopsy.

    The number of biopsy fragments taken will be limited to 5 fragments per target (biopsy site) and the total number of biopsy fragments taken, all targets combined, will be limited to 10 fragments.

- or via surgery performed in the Surgery Department.

The collected fragments will be transferred to the Pathology Department. These will include:

- a fragment to be frozen for storage at the Biological Resources Center (BRC) for the immediate extraction of nucleic acids after verification of tumor cellularity, which will be noted in the report. Only samples with a cellularity greater than 30% will be taken into account. The CRB will be responsible for the storage and extraction of nucleic acids until recovered by the Molecular Oncology and Molecular Oncogenetics laboratories for genomic analysis.
- a fragment fixed and embedded in paraffin will be stored in the Pathology Department for additional immunohistochemical or biological studies.
- at least one fragment to be frozen, after verification of tumor cellularity, for storage in the CRB at -80 °C or in liquid nitrogen (depending on the mode of preservation of the tissue; in a cryotube in nitrogen or a bag at -80 °C) for subsequent proteomic and/or genomic molecular studies.

- specifically, in the cases of patients with locally advanced or metastatic breast cancer and lung cancer: when an additional fragment is available, it will be sent to UMR1068 to be used in an ancillary study validating preclinical therapeutic sensitivity models (xenografts) of locally advanced or metastatic breast tumors (Appendix 3).

In the event that the tumor volume is insufficient to obtain 3 fragments, priority will be given to the fragment intended for the extraction of nucleic acids, followed by the fragment to be fixed and embedded in paraffin. If none of the samples taken allow for the characterization of the tumor's genomic abnormalities, regardless of the stage at which the failure occurs, it will be possible to propose a second biopsy to the patient as long as the inclusion criteria are still met. The decision to take a second sample is at the discretion of the investigator and his team, as is the choice of the metastatic site to sample, which may be the same as or different from the initial sample site.

In the event of a relapse during the follow-up period, the option of proposing a new sampling to the patient for an updated molecular characterization of the tumor is at the discretion of the investigator and his team.

The sample path, from its collection to the Pathology Department, the BRC, and finally the molecular laboratories will be strictly defined and traced for each patient.

4.2.2 – Blood samples

To definitively identify somatic alterations, it is necessary to compare the genome of cancer cells with that of normal cells (e.g. leukocytes) from the same patient. In addition, an analysis of circulating tumor DNA and circulating tumor cells may be performed as part of ancillary studies described in the appendices (Appendices 4 and 5).

For each patient there will be taken, preferably upon inclusion during a check-up, biopsy, or initial visit to an operating room (examination under anesthesia, diagnostic or therapeutic surgical procedure), 5 tubes each with 4 ml of blood in EDTA, i.e. 20 ml of blood for the extraction of DNA from normal cells and analyses of circulating tumor DNA (Appendix 4).

The tubes will be sent to the CRB within 2 hours for centrifugation (at 800G for 10 minutes at 4 °C) and aliquoting (plasma cryotubes for the study of circulating tumor DNA and pellet for DNA extraction) followed by frozen storage. In the event of a relapse during study follow-up, a new blood sample (5 tubes of 4 ml blood in EDTA) may also be taken for analysis of circulating tumor DNA.

Specifically, in cases of patients with breast cancer or cancer of the digestive tract:
As part of the complementary study of circulating tumor cells described in Appendix 5, an additional sample of 5 tubes of 4 ml of blood in EDTA, i.e. an additional 20 ml, will be collected exclusively in patients suffering from locally advanced or metastatic breast cancer or metastatic digestive cancer. The total volume of blood drawn from these patients will be 40 ml.

4.3 – Clinical data

Clinical data from medical files and existing pathology databases of the IPC that were already declared to the CNIL will be recorded in the Case Report Form (CRF).

The anonymized patient data could also be used for the generation of a data warehouse model aiming to both structure clinical and genomic cancer data and set up a system allowing the data to be shared between the different members of a national group (OSIRIS: a national project model on data sharing and integration launched by the Integrated Cancer

Research Sites (SIRICs, French comprehensive cancer centers) and accredited by the French National Cancer Institute).

4.4 – Molecular analyses

The molecular analyses will be carried out by the Molecular Oncology and Molecular Oncogenetics laboratories of the Paoli-Calmettes Institute. Real-time, weekly to bi-monthly analyzes will include testing for DNA copy number alterations and mutations, as well as assessing protein expression. These will concern genes of therapeutic interest that can be targeted by existing drugs or that predict the benefits of a particular systemic treatment. In our experience, it will take between 3 to a maximum of 5 weeks to obtain results (4 weeks in the SHIVA trial for the first 100 patients included). The DNA and RNA will be extracted according to a standard method and their integrity verified on gel and Agilent microarrays.

4.4.1 – Testing for copy number alterations: CGH-array

Gene amplifications and deletions will be identified. Tumor DNA will be hybridized on pangenome arrays for CGH-arrays (244K Agilent Technologies). The choice of microarray may vary depending on technological developments.

4.4.2 – Testing for mutations: targeted next-generation sequencing (NGS)

Mutations in genes of interest (around 370 selected genes, of which nearly 50 are "targetable" by existing drugs) will be identified via targeted next-generation sequencing (NGS). Two different technologies will be used: the PGM Ion Torrent system (Life Technologies) or the MiSeq platform (Illumina), with the HaloPlex kit (Agilent Technologies) including primers for the 370 genes of interest. The relevant medical literature will be monitored during the study in order to complement and update the list of genes coding for proteins of therapeutic interest.

4.4.3 - Validation of protein expression of relevant targets: IHC

Immunohistochemical analysis (IHC) will complement molecular analysis in certain cancers (e.g. hormone receptors and HER2 in breast cancer) and possibly validate the expression of the protein of interest identified via genomic analysis as altered (amplified, mutated). These analyses will be conducted on the fragments fixed and embedded in paraffin in the Pathology Department.

4.4.4 - Bioinformatics analysis of molecular data

The raw files of CGH-array and NGS results will be sent to the Institute's bioinformatics and biostatistics team. The results will then be transmitted in the form of a comprehensive standardized report, which will be recorded in the CRF. The report will be sent to the members of the multidisciplinary molecular tumor board (MTB) for scientific validation before the next meeting. All data generated (molecular analyses, IHC results, etc.) will be stored in a centralized database of the Data Management Unit of the DCRD.

4.4.5 - Complementary molecular analyses.

As part of the secondary objectives of the study, additional molecular analyses will be performed on the remaining tumor DNA, normal DNA, and tumor RNA, such as transcriptome analysis via DNA microarray (24-26) or by RNA-Seq on tumor RNA, or full exome analysis on tumor DNA. Concerning the blood, genomic analyses of circulating tumor DNA will also be performed.

4.5 – Patient care

Patients who are candidates for this project will be given a specialized consultation. During the consultation, the informed consent form will be given to the patient. If the patient agrees to participate in the study (signs consent form), the taking of the samples will be scheduled according to the above description (4.2- biological sampling at inclusion). Once the molecular analyses have been carried out, all the clinical and molecular data will be presented and discussed during a multidisciplinary, weekly, medical/scientific MTB.

This study does not evaluate proposed treatments. Patient care and monitoring will therefore be carried out according to the procedures and practices of the associated center or the specificities of a clinical trial in which the patient could be included, according to the recommendations of the MTB. The patients will then be followed for a period of 3 years. Follow-up data (relapse, death, etc.) will be collected annually. If a cancer susceptibility gene is identified, specific and individualized care will be offered to the patient. This care will not be carried out as part of the study and will require the signing of another specific informed consent form.

V – EVALUATION OF SERIOUS ADVERSE EVENTS

5.1 Definition

Any harmful event occurring in a person participating in biomedical research, whether or not the event is related to the research.

Any event is considered to be a serious adverse event (SAE) if it:

- results in death.
- is life-threatening.
- results in hospitalization of greater than 24 hours or an extension of existing hospitalization.
- causes permanent or a severe temporary disability.
- is the appearance of another cancer.
- causes a birth defect, fetal malformation, or an abortion
- is medically significant.
- is a drug overdose (accidental or intentional).
- is an undesirable clinical or laboratory finding of a serious nature or considered as such by the investigator (with mention of the severity).

An event is not considered a serious adverse event if it:

- results in hospitalization of fewer than 24 hours.

- resulted in a hospitalization scheduled prior to inclusion in the study, as well as any medical procedures included as part of the study (hospitalization for placement of an implant, catheter, etc.) insofar as no complications are observed.

- is an adverse event related to the treatment of the disease.

- is any hospitalization or death related to cancer progression and occurring beyond the notification period defined in the protocol.

5.2 Definition of expected serious adverse events (E-SAE)

This trial does not evaluate treatments proposed following the multidisciplinary consultation meeting. Events described as related to the sampling conducted for the purpose of this study (blood and tumor samples) will be considered as E-SAEs.

The E-SAEs below will not be reported by the sponsor:

- During blood collection: mild pain, the appearance of a bruise on the arm at the site of the blood sample, "discomfort" felt when using a needle, and exceptional physical discomfort.
- During tumor sampling:
    - Percutaneous samples: very moderate risk of hemorrhage, which may require sutures; an exceptional risk of infection.
    - Endoscopic or surgical samples: risks associated with performing the biopsy or surgery (depending on the site and method of sampling), in particular for an operation.

        - <u>Without general anesthesia</u>: pain, moderate risk of hemorrhage post-sampling; moderate risk of thrombosis post-sampling, exceptional risk of infection, pancreatitis, etc.
        - <u>With general anesthesia</u>: risks associated with surgery and anesthesia including a low risk of perforation, which may involve in-hospital monitoring with antibiotic therapy due to the risk of infection, post-operative pain, nausea, post-sampling hemorrhage, post-sampling thrombosis, and in rare cases sepsis, pneumothorax, and pancreatitis.

5.3 Definition of a suspected unexpected adverse reaction (SUSAR)

A SUSAR is an event whose nature, intensity, and course are at odds with the actions performed in this research.

5.4 Course of action in the case of a serious adverse event

The declaration of a serious adverse event occurring within 6 days following a sample will be made within 24 hours of the event to the Department of Clinical Research and Development via the faxing or email of the serious adverse event notification form.

Département de la Recherche Clinique et de l'Innovation
Telephone: +33 04.91.22.37.78
Fax: +33 04.91.22.36.01
email: drci.up@ipc.unicancer.fr

The investigator will note for each event:

- its description, as clearly as possible and according to the medical terminology.
- the start and end dates of the event.
- the measures taken and the need or not for corrective treatment.
- its course that, in the event of a non-fatal event, should be followed until recovery, a return to the previous status, or stabilization of any sequelae.
- the causal relationship between this event and the samples taken as part of the study.
- the causal relationship between the disease being treated and another pathology.

A follow-up report will then be sent to the sponsor to document the evolution of the event by completing the various follow-ups of the initial SAE declaration form.

VI – STATISTICS

6.1 Description of planned statistical methods

Qualitative data will be presented in the form of counts and percentages. The quantitative data will be summarized, with the associated 95% confidence intervals, by the mean, standard deviation, median, and extreme values.

The investigation of the relationship between the histological and clinical variables and the molecular alterations will be performed using chi-square or Fisher, Wilcoxon, or Kruskall-Wallis tests depending on the nature of the data collected.

Overall survival and recurrence-free survival will be evaluated via the Kaplan-Meier method from the date of initial diagnosis and from the date of treatment start post-biopsy. Survival comparisons of different groups of molecular alterations will be performed using log-rank analysis. Prognostic studies on overall survival and progression-free survival will be performed at the end of the study.

6.2 Expected number of research participants to be included

The primary objective is to determine the number of patients whose molecular alteration(s), identified via the samples, can lead to the utilization of targeted therapy. For this purpose, we want to be able to evaluate 300 cases over 3 years. Taking into account a technical failure rate of 35% (found in the SAFIR01 and SHIVA trials), 460 cases will need to be evaluated over 3 years. An intermediate statistical analysis will be performed after the first 100 typed cases to verify the failure rate: if the technical failure rate is greater than 40%, the study will be stopped.

As part of the Substantial Modification Request No.4 and in order to increase the numbers in the patient subpopulations for the performance of the analyzes planned as part of the secondary objectives, it is planned to include 100 additional patients during 1 year, for a total of 560 patients.

The total duration for inclusion is therefore 4 years.

The duration of patient follow-up is 3 years.

The total duration of the study is 7 years.

VII – RIGHT OF ACCESS TO DATA AND SOURCE DOCUMENTS

The investigator guarantees, for both himself and all persons involved during the progress of the trial, the confidentiality of all information provided by the Paoli-Calmettes Institute until the publication of the study's results. This confidentiality obligation will not apply to information that the investigator will be required to communicate to patients as part of their participation in the trial or to already published information.

The investigator undertakes not to publish, disclose, or use, in any way, directly or indirectly, scientific or technical information relating to the study.

However, in accordance with article R 5121-13 of the Public Health Code, the center and the investigator may provide study-related information to:

- the Minister of Health.
- public medical health inspectors.
- pharmacy inspectors.
- ASNM's Director General and inspectors.

The trial may not be the subject of any written or oral comments without the prior approval of the sponsor; all information communicated or obtained during the trial belongs by right to the sponsor, who can freely dispose of it.

VIII – QUALITY CONTROL AND ASSURANCE

The Paoli-Calmettes Institute (IPC) is the sponsor of this project.

The sponsor is responsible for setting up a quality assurance system as described in institutional procedures so that the trial is performed in accordance with both the protocol and Good Clinical Practices.

The IPC's Department of Clinical Research and Development will coordinate the project and ensure quality control.

IX – ETHICAL CONSIDERATIONS

9.1 Regulations

The clinical trial shall be conducted in accordance with:

- the ethical principles of the revised version of the 1964 Helsinki Declaration.
- the Good Clinical Practice (GCP) decision of November 2006.
- the European Directive (2001/20 / EC) on the conduct of clinical trials.
- the Huriet law (No. 88-1138) of December 20, 1988 relating to the Protection of Persons in Biomedical Research and amended by the Public Health Law (No. 2004-806) of August 9, 2004.
- Law No. 2004-801 of August 6, 2004 relating to the protection of physical persons in connection with the processing of personal data and amending law No. 78-17 of January 6, 1978 pertaining to information technology, databases, and civil liberties.
- Law No. 2011-814 of July 7, 2011 relating to Bioethics.

9.2 Ethics Committee

The promoter agrees to submit the study for prior notice to an Ethics Committee (CPP for Comité de Protection des Personnes). The information to be provided will relate to, on the one hand, the methods and nature of the research and, on the other, the guarantees provided for patients participating in this trial.

9.3 Declaration to the competent authorities

This protocol will be the subject of an authorization application to the ANSM.

9.4 Patient informed consent

Prior to carrying out research involving human subjects, the free, informed, and ongoing consent of the subjects must be obtained after they are informed, by the investigator or his representative, of the objective of the research, the conduct and duration of the study, the benefits, the potential risks and constraints of the trial as well as the nature of the product to be studied and the opinion of the Ethics Committee and authorization from ANSM.

The informed consent form will be dated and signed by both the patient and the investigator (one copy to be archived by the investigator and another given to the patient or his legal representative).

The information and informed consent form intended for the patient must be included in the same document in order to avoid any risk of a dispute regarding the content of the disseminated information.

In the case of trials whose objective is to carry out genomics or proteomics analyses, the information form must specify the type of research that will be carried out and the patient must have the possibility of either accepting or refusing the conservation of their biological samples for scientific research purposes.

9.5 Investigators' responsibilities

Investigators involved in the study agree to conduct the clinical trial in accordance with the protocol that will have been approved by the Ethics Committee and the ANSM. Investigators shall not make any modification to the protocol without the sponsor's permission.

The investigator responsible for the trial at each facility shall:

- provide the sponsor with their curriculum vitae as well as those of their co-investigator.
- identify the members of their team participating in the trial and define their responsibilities.
- start patient recruitment after authorization from the sponsor.
- try to include the required number of patients within the established recruitment period.

It is the responsibility of each investigator to:

- obtain each patient's dated and signed informed consent form before any selection procedure specific to the trial.
- regularly fill out the case report forms (CRFs) for every patient included in the trial and give the Clinical Research Assistant (CRA) direct access to source documents so that the latter can validate the CRF data.

- correct, sign, and date any corrections to the CRF of each patient included in the study.
- notify the sponsor of serious adverse events within the required time limits.
- accept regular visits from CRA and possibly from auditors appointed by the sponsor or inspectors from the supervisory authorities.

All documentation relating to the study (protocol, consent forms, CRFs, investigator files, etc.), as well as original documents (laboratory results, radiology images, consultation reports, clinical examination reports, etc.) should be kept in a safe place and treated as confidential material. The archiving of data will be the responsibility of the investigator in accordance with current legislation. The latter must retain the data as well as a patient identification list for a minimum period of 15 years after the end of the study.

9.6 Sponsor's responsibilities

The sponsor is responsible for:

- acquiring insurance covering civil liabilities in the event of harmful consequences of the research for the persons involved.
- communicating to investigators all information necessary for the proper conduct of the research.
- requesting the approval of the Ethics Committee.
- requesting authorization from the ANSM.
- informing the Directors and Pharmacists of participating health care facilities.
- informing the ANSM of any serious events likely to be due to the research or to its early termination the moment the sponsor has been informed.

The sponsor must archive essential documents on study conduct under conditions ensuring their safety for the minimum period stipulated in the GCPs, i.e. 15 years after the end of the research.

X – DATA PROCESSING AND RETENTION OF DOCUMENTS AND DATA RELATING TO RESEARCH

The Data Management Unit of the Paoli-Calmettes Institute will be responsible for processing the study data, which will remain the property of the Paoli-Calmettes Institute, the research's sponsor.

The data processing software used will be the ENNOV CLINICAL® software.

The Paoli-Calmettes Institute agrees to submit this project to the French Data Protection Agency.

XI – CONFIDENTIALITY AND PROPERTIES OF RESEARCH DATA

The investigators agree, for themselves and all persons involved in the trial's progress, to guarantee the confidentiality of all information provided by the Paoli-Calmettes Institute until the publication of the trial's results. This obligation of confidentiality will not apply to information that the investigator will be required to communicate to patients as part of their participation in the trial or to previously published information.

The investigators agree not to publish, divulge, or use, in any way, directly or indirectly, scientific or technical information relating to the trial.

XII – INSURANCE

The IPC agrees to acquire insurance for research involving human subjects in accordance with the current legislation, via the company SHAM (18 rue Edouard Rochet - 69372 LYON CEDEX 08).

XIII – RULES RELATING TO PUBLICATION

The results of this study are the exclusive property of the Paoli-Calmettes Institute and can be used at their sole discretion. All information resulting from the trial is considered confidential, at least until the appropriate analysis and review by the sponsor and the principal investigator has been completed. Results must be published or presented in collaboration with the sponsor.

All publications, abstracts, or presentations including the results of the trial must be submitted for approval to the sponsor (IPC).

In addition, all communications, manuscripts, or presentations must include a section that mentions both the IPC and UMR 1068 as well as any organizations that contributed financially to the research.

The trial's principal investigator will be the primary signatory (1st or last author).

The investigators will be cited according to the number of patients they have or according to the depth of their involvement in the protocol and/or the pathology. The trial's statistician will be cited third, and a member of the DCRD will also be cited.

Likewise, the publication of the ancillary results (biological study) will include the names of those who carried out the ancillary work as well as the names of all the other persons involved.

XIV - BIBLIOGRAPHY

1. Goncalves A, Moretta J, Eisinger F et al. Personalized medicine and breast cancer: anticipatory medicine, prognostic evaluation and therapeutic targeting. **BULL CANCER** 2013;100(12):1295-1310.

2. McDermott U, Downing JR, Stratton MR. Genomics and the continuum of cancer care. **N ENGL J MED** 2011;364(4):340-350.

3. Von Hoff DD, Stephenson JJ, Jr., Rosen P et al. Pilot study using molecular profiling of patients' tumors to find potential targets and select treatments for their refractory cancers. **J CLIN ONCOL** 2010;28(33):4877-4883.

4. Andre F, Bachelot T, Commo F et al. Comparative genomic hybridisation array and DNA sequencing to direct treatment of metastatic breast cancer: a multicentre, prospective trial (SAFIR01/UNICANCER). **LANCET ONCOL** 2014.

5. Le TC, Kamal M, Tredan O et al. Designs and challenges for personalized medicine studies in oncology: focus on the SHIVA trial. **TARGET ONCOL** 2012;7(4):253-265.

6. Tsimberidou AM, Iskander NG, Hong DS et al. Personalized medicine in a phase I clinical trials program: the MD Anderson Cancer Center initiative. **CLIN CANCER RES** 2012;18(22):6373-6383.

7. Roychowdhury S, Iyer MK, Robinson DR et al. Personalized oncology through integrative high-throughput sequencing: a pilot study. **SCI TRANSL MED** 2011;3(111):111ra121.

8. Tran B, Dancey JE, Kamel-Reid S et al. Cancer genomics: technology, discovery, and translation. **J CLIN ONCOL** 2012;30(6):647-660.

9. Bertucci F, Houlgatte R, Nguyen C et al. Gene expression profiling of cancer by use of DNA arrays: how far from the clinic? **LANCET ONCOL** 2001;2(11):674-682.

10. Cornen S, Guille A, Adelaide J et al. Candidate luminal B breast cancer genes identified by genome, gene expression and DNA methylation profiling. **PLOS ONE** 2014;9(1):e81843.

11. Bertucci F, Ueno NT, Finetti P et al. Gene expression profiles of inflammatory breast cancer: correlation with response to neoadjuvant chemotherapy and metastasis-free survival. **ANN ONCOL** 2014;25(2):358-365.

12. Masuda H, Baggerly KA, Wang Y et al. Comparison of molecular subtype distribution in triple-negative inflammatory and non-inflammatory breast cancers. **BREAST CANCER RES** 2013;15(6):R112.

13. Van Laere SJ, Ueno NT, Finetti P et al. Uncovering the molecular secrets of inflammatory breast cancer biology: an integrated analysis of three distinct affymetrix gene expression datasets. **CLIN CANCER RES** 2013;19(17):4685-4696.

14. Bertucci F, Bouvier-Labit C, Finetti P et al. Comprehensive genome characterization of solitary fibrous tumors using high-resolution array-based comparative genomic hybridization. **GENES CHROMOSOMES CANCER** 2013;52(2):156-164.

15. Garcia M, Millat-Carus R, Bertucci F et al. Interactome-transcriptome integration for predicting distant metastasis in breast cancer. **BIOINFORMATICS** 2012;28(5):672-678.

16. Addou-Klouche L, Adelaide J, Cornen S et al. Integrated genomic analysis of breast cancers. **BALKAN J MED GENET** 2012;15(Suppl):71-74.

17. Bertucci F, Bouvier-Labit C, Finetti P et al. Gene expression profiling of solitary fibrous tumors. **PLOS ONE** 2013;8(5):e64497.

18. Bertucci F, Finetti P, Roche H et al. Comparison of the prognostic value of genomic grade index, Ki67 expression and mitotic activity index in early node-positive breast cancer patients. **ANN ONCOL** 2013;24(3):625-632.

19. Bertucci F, Borie N, Roche H et al. Gene expression profile predicts outcome after anthracycline-based adjuvant chemotherapy in early breast cancer. **BREAST CANCER RES TREAT** 2011;127(2):363-373.

20. Bertucci F, Finetti P, Birnbaum D et al. Gene expression profiling of inflammatory breast cancer. **CANCER** 2010;116(11 Suppl):2783-2793.

21. Charafe-Jauffret E, Ginestier C, Bertucci F et al. ALDH1-positive cancer stem cells predict engraftment of primary breast tumors and are governed by a common stem cell program. **CANCER RES** 2013.

22. Birnbaum DJ, Sabatier R, Mamessier E et al. [An "ariditary" form of ovarian cancer]. **MED SCI (PARIS)** 2010;26(12):1040-1042.

23. Birnbaum DJ, Adelaide J, Mamessier E et al. Genome profiling of pancreatic adenocarcinoma. **GENES CHROMOSOMES CANCER** 2011;50(6):456-465.

24. Bertucci F, Finetti P, Cervera N et al. Gene expression profiling shows medullary breast cancer is a subgroup of basal breast cancers. **CANCER RES** 2006;66(9):4636-4644.

25. Bertucci F, Finetti P, Rougemont J et al. Gene expression profiling for molecular characterization of inflammatory breast cancer and prediction of response to chemotherapy. **CANCER RES** 2004;64(23):8558-8565.

26. Finetti P, Cervera N, Charafe-Jauffret E et al. Sixteen-kinase gene expression identifies luminal breast cancers with poor prognosis. **CANCER RES** 2008;68(3):767-776.

27. Ginestier C, Hur MH, Charafe-Jauffret E et al. ALDH1 is a marker of normal and malignant human mammary stem cells and a predictor of poor clinical outcome. **CELL STEM CELL** 2007;1(5):555-567.

28. Schwarzenbach H. Circulating nucleic acids as biomarkers in breast cancer. **BREAST CANCER RES** 2013;15(5):211.

29. Wang S, Zheng G, Cheng B et al. Circulating Tumor Cells (CTCs) Detected by RT-PCR and Its Prognostic Role in Gastric Cancer: A Meta-Analysis of Published Literature. **PLOS ONE** 2014;9(6):e99259.

30. Hodgkinson CL, Morrow CJ, Li Y et al. Tumorigenicity and genetic profiling of circulating tumor cells in small-cell lung cancer. **NAT MED** 2014.

31. van de Stolpe A, den Toonder JM. Circulating Tumor Cells: What Is in It for the Patient? A Vision towards the Future. **CANCERS (BASEL)** 2014;6(2):1195-1207.

32. Krebs MG, Metcalf RL, Carter L et al. Molecular analysis of circulating tumour cells-biology and biomarkers. **NAT REV CLIN ONCOL** 2014;11(3):129-144.

33. Dobrila-Dintinjana R. Circulating cancer cells are potential weapon for future generations. **HEPATOGASTROENTEROLOGY.** 2014;61(129):5-8.

34. Carter L, Metcalf R, Blackhall FH et al. Biology and clinical relevance of circulating tumour cells. **J THORAC DIS** 2012;4(5):453-455.

35. Smerage JB, Barlow WE, Hortobagyi GN et al. Circulating Tumor Cells and Response to Chemotherapy in Metastatic Breast Cancer: SWOG S0500. **J CLIN ONCOL** 2014.

36. Normanno N, Rossi A, Morabito A et al. Prognostic value of circulating tumor cells' reduction in patients with extensive small-cell lung cancer. **LUNG CANCER** 2014.

37. Seeberg LT, Waage A, Brunborg C et al. Circulating Tumor Cells in Patients With Colorectal Liver Metastasis Predict Impaired Survival. **ANN SURG** 2014.

38. Lalmahomed ZS, Mostert B, Onstenk W et al. Prognostic value of circulating tumour cells for early recurrence after resection of colorectal liver metastases. **BR J CANCER** 2015;112(3):556-561.

39. Mostert B, Sieuwerts AM, Bolt-de VJ et al. mRNA expression profiles in circulating tumor cells of metastatic colorectal cancer patients. **MOL ONCOL** 2015;9(4):920-932.

40. Huang X, Gao P, Song Y et al. Relationship between circulating tumor cells and tumor response in colorectal cancer patients treated with chemotherapy: a meta-analysis. **BMC. CANCER** 2014;14:976.

41. Sotelo MJ, Sastre J, Maestro ML et al. Role of circulating tumor cells as prognostic marker in resected stage III colorectal cancer. **ANN ONCOL** 2015;26(3):535-541.

42. Poruk KE, Valero V, III, Saunders T et al. Circulating Tumor Cell Phenotype Predicts Recurrence and Survival in Pancreatic Adenocarcinoma. **ANN SURG** 2016.

43. Gorner K, Bachmann J, Holzhauer C et al. Genetic analysis of circulating tumor cells in pancreatic cancer patients: A pilot study. **GENOMICS.** 2015;106(1):7-14.

44. Kaifi JT, Kunkel M, Das A et al. Circulating tumor cell isolation during resection of colorectal cancer lung and liver metastases: a prospective trial with different detection techniques. **CANCER BIOL THER.** 2015;16(5):699-708.

XV – APPENDICES

Appendix 1: ECOG performance scale
Appendix 2: Investigations summary
Appendix 3: Validation of preclinical models of therapeutic response in locally advanced or metastatic breast tumors
Appendix 4: Study of circulating tumor DNA
Appendix 5: Study of circulating tumor cells

Appendix 1: ECOG (Eastern Cooperative Oncology Group) performance scale

ECHELLE	ETAT GENERAL<br>ECOG-ZUBROD/WHO
0	Fully active, able to carry on all pre-disease performance without restriction.
1	Restricted in physically strenuous activity but ambulatory and able to carry out work of a light or sedentary nature, e.g., light housework, office work.
2	Ambulatory and capable of all self-care but unable to carry out any work activities; up and about more than 50% of waking hours.
3	Capable of only limited self-care; confined to bed or chair more than 50% of waking hours.
4	Completely disabled; cannot carry on any self-care; totally confined to bed or chair.

<u>Appendix 2: Investigations summary</u>

<table>
<tr><td colspan="2">Samples</td></tr>
<tr><td colspan="2">Tumor samples</td></tr>
<tr><td>Biopsy
(Sampling limited to 5 fragments per target and to a total of 10 fragments maximum, all targets combined)

or

Surgical specimen</td><td>Send to the Pathology Department within 2 hours.
At least 3 fragments, in order of priority:
- A fragment, after verification of tumor cellularity, to be frozen for the imminent extraction of nucleic acids.
- A fragment fixed and embedded in paraffin for complementary immunohistochemical or biological studies.
- At least one fragment, after verification of tumor cellularity, to be frozen for use in subsequent molecular studies of proteomics and/or genomics.</td></tr>
<tr><td colspan="2">Blood samples</td></tr>
<tr><td>5 tubes of 4 ml blood in EDTA, i.e. 20 ml to obtain the genome of normal cells and to perform analyses on circulating tumor DNA.</td><td>Send to Biological Resource Center within 2 hours.

Centrifuge: 800G for 10 minutes at 4°C. Separate:
- the pellet
Freeze the dried pellet for nucleic acid extraction.
- the plasma
Aliquot in cryotubes and store at -80°C for analyses of circulating tumor DNA.</td></tr>
</table>

<table>
<tr><td colspan="2">Supplementary samples</td></tr>
<tr><td colspan="2">Tumor samples in the specific case of breast cancer (locally advanced or metastatic) or lung cancer</td></tr>
<tr><td>Biopsy

or

Surgical specimen</td><td><u>When available</u>, an additional fragment will be transferred, via the Pathology Department, to the Marseille Cancer Research Center (UMR1068) for use in the ancillary project validating predictive preclinical models of therapeutic response.</td></tr>
<tr><td colspan="2">Blood samples in the specific case of breast or digestive cancer</td></tr>
<tr><td>5 tubes of 4 ml in EDTA (20 ml total) for analyses of circulating tumor cells.</td><td>Send while still cool to the central reception of the IPC and then to the Marseille Cancer Research Center (UMR1068) for the isolation of circulating tumor cells.</td></tr>
<tr><td colspan="2">Tumor and blood samples in the event of a relapse (at the discretion of the investigator)</td></tr>
<tr><td>Biopsy
(Sampling limited to 5 fragments per target and a maximum of 10 fragments total, all targets combined)</td><td>Send to Pathology Department within 2 hours.
At least 3 fragments, in the following order of priority:
- A fragment, after verification of tumor cellularity, to be frozen for the imminent</td></tr>
</table>

or<br><br>Surgical specimen	extraction of nucleic acids.<br>- A fragment fixed and embedded in paraffin for complementary immunohistochemical or biological studies.<br>- At least one fragment, after verification of tumor cellularity, to be frozen for use in subsequent molecular studies of proteomics and/or genomics.
5 tubes of 4 ml in EDTA (20 ml total) for analyses of circulating tumor cells.	Send to Biological Resource Center within 2 hours.<br><br>Centrifuge: 800G for 10 minutes at 4°C.<br>Aliquot in cryotubes and store at -80°C for analyses of circulating tumor DNA.

Appendix 3: Validation of preclinical models of therapeutic response in locally advanced or metastatic breast tumors

Our team has demonstrated the feasibility of establishing patient-derived xenografts from primary breast cancer tumors, which maintain their essential clinical, pathological, and molecular characteristics and allow for the possibility of their use in developing targeted personalized therapies (21). In this project, we aim to establish a bank of breast cancer metastases.

This study aims to validate the feasibility of using these models in vivo and in vitro as predictive models of therapeutic response in patients with metastatic breast cancer.

Establishment of a patient-derived xenograft bank

The metastatic samples obtained via biopsy or surgery will be dissociated by mechanical and enzymatic methods (digestion by collagenase/hyaluronidase) to generate unicellular suspensions of tumor cells.

These tumor cells are then orthotopically implanted in the fat pads of NSG mice as described by Ginestier et al. 2007 and Charafe-Jauffret et al. 2013 (21, 27). Tumor growth is then monitored bi-weekly, and the xenografted tumor compared to the primary tumor histologically, phenotypically, and molecularly at early or later passages to detect and prevent model drift.

Establishment of an organoid bank

Over the past 10 years, a large number of studies have contributed to the evolution of cancer modeling systems, from the traditional two-dimensional cancer cell culture system to more complex three-dimensional (3D) models. Because of this, we now know that a tumor is not just a simple mass of cancerous cells that proliferate, but rather is a complex tissue composed of a dynamic extracellular matrix with a stromal component and immune and endothelial cells. Significant efforts are currently being made to better recapitulate the tumor microenvironment, as well as discussing the potential of tumor engineering. These approaches include the development of modified matrices and 3D co-cultures to reproduce the complexity of tumor-stroma interactions in vitro and are the first step towards ex-vivo tumor modeling.

Starting from tumor stem cells, it is now possible to generate tumor organoids that better recapitulate certain aspects of the original tumor, much more so than in simple 2D cultures, as well as more quickly (< 1 month) and with greater efficiency (> 75% regardless of tumor subtype) than xenograft models. Therefore, generating organoids from tumors could be a very relevant alternative, making it possible to amplify tumor material in order to generate a bank of tumor organoids in sufficient quantity, at a lower cost, and within a timeframe compatible with trials of therapeutic molecules, such as in a personalized medicine study like the Permed study.

Study design:

First, familiarization with generating breast tumor organoids, all subtypes:

1- Establishment of one or more environments suitable for different sub-types of breast cancer:
   a. Analyses performed on patient tumors amplified beforehand from xenografts.

b. Validation in patients included prospectively.
2- Establishment of a growth (monitoring the growth of generated organoids) and heterogeneity score:
   a. Rate
   b. Shape
   c. Survival and passages, presence of stem cells
3- Comparison with the existing xenograft model, taking into account the subtypes and similarity to the initial tumor.
4- Presence or not of mutations identified in the tumor.
5- Evaluation of using organoids for functional tests, study of the tumor microenvironment, testing targeted therapies (antibodies, etc.), evaluating metastatic potential (quantification of the strain's potential, migratory capacities, invasive properties, etc.).

Secondly, the creation of a breast cancer organoid bank (frozen organoids). This bank can be used to carry out functional analyses of well-characterized tumors and analyses involving targeted therapies may also be developed.

Assessment of therapeutic response

After obtaining and expanding the xenograft, the mutational status will be assessed as well as the status of the patient's tumor sample.

The tumor cells obtained via the mouse model will be cultivated in vitro for a study of therapeutic responsiveness as a "short-term culture".

These cells will then be exposed in vitro for 96 hours to a panel of drugs containing nearly 50 molecules including chemotherapies, targeted therapies, and innovative products.

The drugs considered to be "active" in vitro will be validated in vivo, in mice.

Additional analyses based more specifically on treatment effectiveness in cancer stem cells, as well as identifying anti-CSC treatments, could be carried out using this model.

Appendix 4: Study of circulating tumor DNA

Tumor development is often accompanied by increased levels of tumor DNA circulating in the blood.

In this context, the release of DNA into the bloodstream may be caused by the primary tumor, circulating tumor cells, distant micrometastases, and normal cells such as hematopoietic and stromal cells (28). While this circulating tumor DNA can now be detected, its usefulness as a non-invasive diagnostic biomarker ("liquid biopsy") reflecting the characteristics of the primary tumor and micrometastases remains to be demonstrated.

Our study aims to assess the interest and feasibility of sequencing circulating tumor DNA compared to sequencing tumor samples.

The presence of circulating tumor DNA will be evaluated from the plasma obtained from the 4 blood tubes in EDTA.

The tubes will be centrifuged at 800g for 10 minutes. The plasma will then be aliquoted and frozen in cryotubes at -80°C.

When thawing a series of samples, the following protocol will be adhered to:

- Centrifugation at 20,000g for 10 minutes.
- Recovery of the supernatant and addition of 200 µL of Proteinase K.
- Addition of the ACL buffer containing the RNA carrier.
- Incubation for 30 minutes at 60°C.
- Addition of 3 ml of ACB buffer to the lysate - vortex 30 seconds.
- 5 minutes in ice.
- Depositing of the lysate in extension tubes for the QIAamp column on the QIA Vac 24 Plus vacuum system.
- Creation of a vacuum (800 mbar).
- Once the column is dry, addition of washing buffer 1, then buffer 2, then pure ethanol.
- Once the column is dry, centrifugation at 20,000g for 3 minutes.
- Drying of the column for 10 minutes at 56°C.
- Application of the elution buffer, and after 5 minutes, centrifugation for 1 minute at 20,000g for nucleic acid elution.

The molecular analyses will be carried out under the same conditions as those described in the protocol (section 4.4-molecular analyses).

Appendix 5: Study of circulating tumor cells

Circulating tumor cells (CTCs) are exploding as a new major prognostic biomarker that could play an essential role in the implementation of targeted therapies for cancers and their metastatic evolution (29-31).

Although the existence of CTCs, as precursors of metastatic lesions, was suggested over 100 years ago, formal evidence that CTCs are indeed capable of initiating metastatic lesions has not been demonstrated due to major technical limitations that have hitherto prevented their precise genomic and phenotypic characterization (32, 33). Indeed, CTCs are rare events detected in approximately 20 to 50%, depending on the cancer, of metastatic patients (34). The detection of CTCs in the blood is associated with a poorer prognosis, reduced survival, may reflect an lack of treatment response (or a partial response that would target sensitive clones while resistant clones remain), or may even predict the non-resectability of a tumor in the cases of the most aggressive tumors (35-37).

CTCs as reflections of intratumoral clonal heterogeneity with invasive potential must be studied in order to improve their targeting, depending on their stage of development in tumorigenesis.

Our objectives for this study include:

- carrying out a preliminary study in order to demonstrate that we have, at the Paoli-Calmettes Institute, the tools necessary to further explore the characterization of these biomarkers.
- identifying molecular alterations on the purest CTC in parallel to the primary tumor and/or metastasis in order to assess intratumoral molecular heterogeneity and establish disrupted signaling pathways and potential therapeutic targets.

<u>Pilot phase: Implementation of a new technique for isolating CTCs</u>

The isolation of CTCs is usually performed via the CellSearch® system, which is based on the epithelial cell marker anti-Epcam. However, CTCs decrease or lose the expression of this marker when passing into circulation, thus leading to an underestimation of the real number of circulating cells.

We propose here an original approach in two steps, utilizing a volume of 16 ml of blood in EDTA:

1. Enrichment by way of negative selection where all the hematopoietic cells are removed, leaving only those cells that are not usually found in the area.
2. Further purification of the previously enriched cells, including the detection of molecules associated with the mesenchymal-epithelial transition process, as well as new markers of carcinogenesis (MUC1, MUC4, etc.). These cells will be selected by flow cytometry, deep array (phenotyping/genotyping) or any other suitable technique, depending on the availability of the devices which are being/will be used. The purity of these cells will be assessed by evaluating the expression of transcripts of typical epithelial markers, such as cytokeratins.

The first part of this study will be carried out, initially, using samples obtained from:

- 20 patients with locally advanced breast cancer or, if the results of the approach are conclusive, all patients with breast cancer included in the PERMED01 study. In the absence of conclusive results, the study will be stopped.

– 20 patients with digestive cancer (especially pancreatic cancer). Indeed, these cancers, by virtue of their clinical characteristics, appear to be good candidates for studying CTC detection sensitivity (38-41).

In the specific case of pancreatic cancer, two studies recently confirmed the prognostic role of CTCs (42, 43).

In addition, Kaifi et al. recently compared two different isolation techniques: CellSearch® (25 samples) and a filtration technique (16 samples), and demonstrated that CTCs are, in the majority of cases, undetectable with CellSearch® but almost always detectable using the filtration technique (44). We would like to assess the sensitivity limits of our detection tools, in particular our filtration technique, which is similar to that described by Kaifi et al. We hope to be able to detect CTCs in at least 10 patients.

<u>Study of genomic, transcriptomic and mutatome profiles (by array-CGH, transcriptome analysis, and NGS)</u>

The objective of this program will ultimately be to obtain genomic, transcriptomic and mutatome profiles (by array-CGH, transcriptome analysis, and NGS) from a virtually pure population of CTCs and/or a single cell. These analyses will be performed in the Molecular Oncology laboratory (UMR1068) using a targeted panel for analysis via high throughput sequencing data and/or transcriptomic data, in order to study the major genes involved in the tumor aggressiveness and drug susceptibility. These analyses will be carried out under the same conditions as those described in the protocol (section 4.4-molecular analyses).

<u>Dissemination phase</u>

The feasibility study demonstrated the effectiveness of the CTC purification procedure (detection in all patients, with mean = 52.25 CTC/ml; Std Dev = 84.04) up to the cultivation step. These results confirm the feasibility of the technique that we have developed within the framework of the pilot phase.

However, the number of CTCs was not associated with growth in vitro, suggesting that, more than the count, other descriptive parameters are probably more important to take into account, notably the number of clusters of CTCs.

We will search for correlations between these parameters and patient prognostic data.

For the dissemination phase, we propose to extend this study by collecting 5 tubes of blood in EDTA (i.e. an additional tube compared to the pilot phase), which will be used:

1. in the sequencing and phenotyping of CTCs (E Mamessier group, D Birnbaum team, UMR1068, Marseille Cancer Research Center).
2. for the culture and characterization of CTC (E Mamessier group, D Birnbaum team, UMR1068, Marseille Cancer Research Center).

The purified viable CTCs will be cultured under hypoxic conditions in a suitable culture medium for these rare cells (Cayrefour et al. Cancer Research 2015, Yu et al. Science 2014). This will permit cell growth over a few weeks and will be capable of producing sufficient material to study their migratory and invasive properties in experimental models in vitro.

These functional analyses will aim to identify the molecular mechanisms responsible for degrading the extracellular matrix, in particular via specialized machinery: invadopodia. Both

data from the literature and our preliminary results indicate that the microtubule cytoskeleton and associated proteins are involved in tumor invasion (Kikushi et al. Cancer Science 2008, Schoumacher et al. Journal Cell Biology 2010).

3. for the study of invasive markers (A Badache team, CM Research Center, UMR1068)

In addition, we are implementing systematic approaches for identifying new components of invadopodia. The team involved in this project (A Badache, CRCM, UMR1068) have expertise in the study of cell migration and will study the contribution of microtubules and associated proteins, as well as the molecular components of invadopodia, to the invasive properties of CTCs. These molecules could represent new markers of invasion and metastatic progression, or even specific longer-term therapeutic targets.

The final objective of this study is to identify molecular markers involved directly or indirectly in the ability of CTCs to invade target tissues from the bloodstream. The functional data will be compared with clinical data as well as sequencing and phenotyping data from samples from these same patients.
