# Supplementary material for: Prospective high-throughput genome profiling of advanced cancers: results of the PERMED-01 clinical trial
Source: Genome Med. 2021 May 18;13:87. doi: 10.1186/s13073-021-00897-9 (PMC8132379; doi:10.1186/s13073-021-00897-9)
Supplement: Supplementary file 2 — Additional file 2: Supplementary methods. Contains additional methods regarding the trial design, the genome analyses, the molecular precision oncology report and molecular tumor board, and the statistical analyses. [file 13073_2021_897_MOESM2_ESM.docx]

**Additional file 2. Supplementary methods**

This file (.doc file) contains additional methods regarding the trial design, the genome analyses, the molecular precision oncology report and molecular tumor board, and the statistical analyses.

**METHODS**

**Study objectives and design**

PERMED-01 was a prospective unicentric clinical trial promoted by and conducted at the Paoli-Calmettes Institute (Marseille, France). Its primary objective was to evaluate the number of patients with locally advanced or metastatic cancer for whom identification of actionable genetic alterations (AGAs) in tumor samples using t-NGS and aCGH could lead to the delivery of a “matched therapy”. Secondary objectives herein reported included a description of patients with AGA who received a “matched therapy” and their clinical outcome and comparison of AGA identification with t-NGS and aCGH *versus* whole-exome sequencing (WES). Additional secondary objectives, that have been or will be reported elsewhere, included the description of molecular alterations of advanced solid cancers and their relationship with the clinicopathological characteristics, including progression-free survival and overall survival, their comparison with molecular alterations of the paired primary tumor if available, pan-genomic molecular analysis of metastatic samples with full exome sequencing [[1](#_ENREF_1)] and transcriptome analysis, analysis of circulating tumor DNA, analysis of circulating tumor cells (for breast and digestive cancers), and development of preclinical models for prediction/analysis of tumor response/resistance (xenografts, short-term culture, and organoids for breast cancer). Inclusion criteria were age ≥18 years, pathological diagnosis of solid cancer, locally advanced or metastatic stage progressive during at least one line of prior therapy and with an accessible lesion for biopsy, Eastern Cooperative Oncology Group (ECOG) Performance Status ≤2, affiliation to Social Insurance, and signed informed patient’s consent for participation. Exclusion criteria were symptomatic or progressive leptomeningeal or brain metastases, bone or brain metastasis as sole metastatic site, pregnancy or breastfeeding, and person in an emergency situation or subject to a measure of legal protection or unable to express consent. The study, registered as ClinicalTrials.gov identifier NCT02342158, was approved by the French National Agency for Medicines and Health Products Safety, a national ethics committee (CPP Sud-Méditerranée I), and our Institutional Review Board. It was conducted in accordance to the Good Clinical Practice guidelines of the International Conference on Harmonization. All patients gave their informed consent for inclusion, biopsy, and genomic analysis. Once enrolled, a new tumor biopsy or resection was proposed to the patient. The study was reported according to the CONSORT checklist.

***Biopsy and genome analysis***

All genomic analyses were done on *de novo* biopsies, and not archival samples. After having given signed informed consent, the patient underwent tumor biopsy - either visually, radiology-guided or surgical – or a tumor resection. Samples were divided in several parts, including one that was fixed and paraffin-embedded for diagnosis confirmation and standard immunohistochemistry (IHC), and one that was frozen after assessment of tumor cellularity by the pathologist. Only frozen samples with at least 30% of tumor cells were retained for nucleic acids extraction and genomics analysis. Tumor DNA and germline DNA from whole-blood samples (when available) were extracted, controlled on Agilent Bioanalyzer (Agilent Technologies, Massy, France) and concentration assessed by using Qubit dsDNA BR Assay [[2](#_ENREF_2)]. The genomic profiles were established by using aCGH and t-NGS.

aCGH was done from 500 ng of DNA onto high-resolution 4×180K CGH microarrays (SurePrint G3 Human CGH Microarray Kit, Agilent Technologies, Massy, France) as previously described [[2](#_ENREF_2)]. Human female or male DNA was used as reference (G1521 or G1471 Promega, respectively). All probes for aCGH were mapped according to the hg19/NCBI human genome mapping database. Analysis of copy number alterations (CNA) was limited to the 802 genes present in at least of one NGS panel. The copy-number was estimated for each gene by taking the value of the segment with the highest amplitude, then categorized into amplification (Log_2_ratio >1), and deletion (Log_2_ratio <-1). The gains and losses were not considered. The array-CGH data from this trial are available in the ArrayExpress database at EMBL-EBI (www.ebi.ac.uk/arrayexpress) under the E-MTAB-9998 accession number. For each tumor, a Homologous Recombination Deficiency score (HRD_aCGH_ score), based on losses of heterozygosity (LOH), was calculated from all tested aCGH genes as previously reported [[3](#_ENREF_3)]: a score ≥10 was considered as HRD-high.

t-NGS was done from 200 ng of DNA as previously described [[2](#_ENREF_2)]. Four chronologically extended custom-made panels of “cancer-associated” genes selected for their involvement in cancers were used (**Table S1**): V7, V8, V10, and V11 covering 395, 494, 560, and 795 genes, respectively. More than 93% of samples were profiled using V8, V10 or V11. In order to identify patients with a suspected hereditary cancer predisposition, these panels included from 49 to 67 cancer predisposition genes commonly analyzed by the BROCA Cancer Risk panel (https://testguide.labmed.uw.edu/public/view/BROCA). The DNA libraries of all coding exons and intron-exon boundaries of genes were prepared using the HaloPlex Target Enrichment System (Agilent, Santa Clara, CA, USA) as previously described [[2](#_ENREF_2)]. Sequencing was done using the 2x150-bp paired-end technology on the Illumina MiSeq and NextSeq500 platforms (Illumina, San Diego, CA, USA). Tumor samples and matched normal samples (available for 315 patients) were sequenced at respective median depths of 732x and 387x. Sequence data were aligned to the human genome (UCSC hg19) and alignment processed as previously described [[4](#_ENREF_4)]. Variants calling and annotation were done as previously described [[5](#_ENREF_5)]. Somatic variants calling were done with SomaticSeq [[6](#_ENREF_6)]. The raw NGS data from this trial are available in the European Genome-phenome Archive (EGA: accession EGAS00001004554). The tumor mutational burden (TMB) and MSI-H status were defined in the 295 tumors with a matched normal sample sequenced. TMB was defined by the number of somatic coding mutations including missense, nonsense, silent, and indel divided by the panel size [[7](#_ENREF_7)], defined by the base number of the coding gene regions. The threshold for TMB-high was 10 mutations/Mb as recommended [[8](#_ENREF_8)]. Microsatellite instability detection was done using the software MSIsensor [[9](#_ENREF_9)] that computes a “MSI score” reflecting the amount of replication slippage variants at microsatellite regions. We used a cut-off of 10% to detect MSI-H tumors.

Whole-exome sequencing (WES) data were available for 112 pairs of metastatic breast cancer and matched-germline DNA previously profiled using Illumina© technology [[1](#_ENREF_1)] allowing the comparison of AGAs, HRD score and TMB (EGA repository: accession EGAS00001003290 (https://ega-archive.org/studies/EGAS00001003290). The HRD score was measured from WES data (HRD_WES_) as described [[10](#_ENREF_10)], by compiling the three independent measures of genomic instability: number of LOH, number of telomeric-allelic imbalances (TAI), and number of large-scale state transitions (LST), scored from FACETS results. The score was the sum of TAI, LST and LOH scores. The profile was considered as HRD-high when the HRD_WES_ score was ≥42 [[11](#_ENREF_11)]. Regarding the TMB, the comparison was done using both continuous and binary values.

**Molecular precision oncology report and molecular tumor board**

All molecular alterations identified by aCGH and t-NGS were reviewed one by one by two molecular genomists responsible for generating a molecular report describing the retained molecular alterations, AGAs, and eventual cancer-predisposing genetic alterations. This report was discussed during our weekly institutional molecular tumor board (MTB) to recommend and prioritize possible matched therapy. Actionability of alterations was defined by our MTB experts in real-time. First, the following alterations were considered. For oncogenes, we retained the focal gene amplifications (6 or more copies), hotspot mutations activating or associated with therapeutic resistance, and mutations with undescribed pathogenic effect but with characteristics that may suggest a pathogenic effect (kinase domain or other functionally-important protein domain). For tumor suppressor genes, we considered the following loss-of-function alterations: homozygous deletion, heterozygous deletion associated with a loss of expression (IHC), heterozygous deletion associated with a known inactivating mutation, and heterozygous deletion associated with a mutation with undescribed pathogenic effect (but with characteristics that may suggest a pathogenic effect: nonsense mutation or frameshift insertion/deletion of one or more nucleotides introducing a stop codon or destabilizing the protein structure). Second, the actionability of an alteration was defined by our MTB experts in real-time by existence of a drug targeting the altered protein, either directly or indirectly by impacting the activated pathway. The biomarker/treatment association was estimated by using OncoKB [12] (by considering all evidence levels, from 1 to 4) and/or the clinical or pre-clinical data from the literature (suggesting a link with therapeutic response or resistance) and/or the existence of a clinical trial requiring alteration for enrollment.” AGAs were represented by single-gene alterations or by high HRD or TMB, and MSI-H. The recommended therapy was defined as “matched” when its prescription was based upon an AGA identified uniquely thanks to the PERMED-01 molecular screening. Otherwise, it was defined as “non-matched therapy”. Of note, this definition was independent from EMA approval at the time of treatment initiation. Treatment assignment was not randomized but at the discretion of physician and patient. The patients actually initiating a systemic treatment, “non-matched” or “matched” according to the MTB proposal, were monitored for tumor response according to the clinical trial in which they were enrolled or to the routine medical practice. When possible cancer susceptibility was identified, the result was explained to the patient during a consultation with an oncogenetist.

**Statistical analysis**

In order to have a sufficient number of patients with different cancers and with an identifiable AGA, we wanted to evaluate 300 patients enrolled over three years. Previous studies 13,14] had reported a 35% technical failure rate. Thus, we initially planned to include 460 patients. On November 2017 after three years of inclusion and inclusion of the 460^th^ patient, and in order to increase the numbers in certain patients subpopulations for certain secondary objectives, we asked the French regulatory authorities to amend the protocol to allow enrollment of 100 additional patients over 1 year. The protocol was amended and the trial was reopened to inclusions on September 2018. On September 2019, after one year of inclusion and enrollment of 90 additional patients, the trial was closed. Thus a total of 550 patients had been enrolled between November 2014 and September 2019, over less than 4 years of inclusion (November 2014 to November 2017, then September 2018 to September 2019). An intermediate analysis was planned at the 100^th^ patient with a stopping rule if 40% or more of technical failures were observed.

Baseline patient and disease characteristics were summarized using descriptive analysis by using counts and frequencies for categorical variables and medians (ranges) for continuous variables. Differences among the groups were assessed using the Fisher exact test for categorical variables, and Mann-Whitney test for continuous variables. The primary endpoint was the number of patients with AGAs prospectively identified in real-time in tumor samples. A retrospective *post-hoc* analysis of this endpoint was added using a more stringent AGA definition based on the last OncoKB version (v2.10). Secondary endpoints herein reported included a description of clinicopathological characteristics of patients with AGA who received a “matched therapy” *versus* “non-matched therapy”, including their clinical outcome, and a comparison of AGA identification with t-NGS and aCGH *versus* WES. The main efficacy endpoint was the PFS2/PFS1 ratio, defined as the ratio of progression-free survival (PFS2) on treatment given after molecular testing (therapy 2) to the PFS on the immediate previous treatment (PFS1, therapy 1): this ratio allows intra-patient comparisons in the precision medicine trials by exploring the benefit of a “matched therapy” relative to the previously received treatment [[15](#_ENREF_14)]. PFS was defined as the time from start of treatment to progression for PFS1 and to progression or death from any cause for PFS2. Patients without any event were censored at the date of last contact. A ratio ≥1.3 is considered as a non-ambiguous sign of activity for the new treatment, relative to previously received treatments [[15](#_ENREF_14)]. The patients with missing PFS2 or PFS1, and those under therapy 2 and non-progressing after duration inferior to 1.3-fold the PFS1 were excluded from analysis. Exploratory *post-hoc* analysis searched for clinical parameters associated with ratio ≥1.3 among the patients with AGA and treated: patients’ age, sex, ECOG Performance Status (PS), cancer type, pathological type, extension stage, number of metastatic sites, and number of previous lines of chemotherapy; ESCAT clinical evidence levels of the matched therapy/AGA pair assessed at the end of study by two clinician authors blinded to patient outcome [[16](#_ENREF_15)], and the therapeutic class were added in analysis dedicated to the 94 patients with “matched-therapy”. Three variables were included as continuous variables (patients’ age, number of metastatic sites, and number of previous lines of chemotherapy), whereas other variables were included as categorical variables. Analyses were done using Fisher’s exact test and Mann-Whitney for differences in proportions and medians respectively, and with logistic regression analyses (glm) with and without adjustment upon the cancer type (breast cancer *versus* non-breast cancer, given the imbalance in favor of breast cancer) and with and without correction for multiple testing using the Benjamini-Hochberg method [[17](#_ENREF_16)] for controlling the false discovery rate (FDR). Other efficacy endpoints included the response rate, PFS2, and overall survival (OS). The response was considered as assessable when the patient had received at least 8 weeks of treatment, and was radiologically assessed according to Response Evaluation Criteria In Solid Tumors (RECIST version 1.1) and scheduled according to the local guidelines for each disease. OS was measured from the date of start of treatment for post-biopsy progression until death or date of last news. The follow-up was calculated from the date of biopsy to the date of last news for patients alive. Probabilities of PFS and OS were estimated using the Kaplan-Meier method and univariate associations were evaluated with the log-rank test. The comparison of efficacy endpoints between the “matched therapy” and “non-matched therapy” groups was also done using regression analyses (glm and Cox proportional hazards models) with adjustment upon the cancer type (breast cancer *versus* non-breast cancer) and FDR correction. The comparison of results obtained with t-NGS/aCGH and WES were done using the McNemar χ^2^ test and Cohen Kappa coefficient. According to the French law, this interventional research protocol also planned to collect the serious adverse events related to the biological sampling procedures and occurring within the seven days after; theywere graded according to the NCI-CTCAE (version 3.0). Exploratory post-hoc analyses were done for the primary endpoint and for one secondary endpoint (PFS2/PFS1). Statistical analyses were done either with the R software version 2.15 or Prism software (Graphpad software, San Diego, CA, USA) and the significance level was set to 5%. Except for primary analysis, all tests were two-sided.

**References**

1. Bertucci F, Ng CKY, Patsouris A, Droin N, Piscuoglio S, Carbuccia N, Soria JC, Dien AT, Adnani Y, Kamal M, et al. Genomic characterization of metastatic breast cancers. Nature. 2019,569:560-4.

2. Tyran M, Carbuccia N, Garnier S, Guille A, Adelaide J, Finetti P, Toulzian J, Viens P, Tallet A, Goncalves A, et al. A Comparison of DNA Mutation and Copy Number Profiles of Primary Breast Cancers and Paired Brain Metastases for Identifying Clinically Relevant Genetic Alterations in Brain Metastases. Cancers (Basel). 2019,11.

3. Abkevich V, Timms KM, Hennessy BT, Potter J, Carey MS, Meyer LA, Smith-McCune K, Broaddus R, Lu KH, Chen J, et al. Patterns of genomic loss of heterozygosity predict homologous recombination repair defects in epithelial ovarian cancer. Br J Cancer. 2012,107:1776-82.

4. Bertucci F, Finetti P, Guille A, Adelaide J, Garnier S, Carbuccia N, Monneur A, Charafe-Jauffret E, Goncalves A, Viens P, et al. Comparative genomic analysis of primary tumors and metastases in breast cancer. Oncotarget. 2016,7:27208-19.

5. Xerri L, Adelaide J, Avenin M, Guille A, Taix S, Bonnet N, Carbuccia N, Garnier S, Mescam L, Murati A, et al. Common origin of sequential cutaneous CD30+ lymphoproliferations with nodal involvement evidenced by genome-wide clonal evolution. Histopathology. 2019,74:654-62.

6. Fang LT, Afshar PT, Chhibber A, Mohiyuddin M, Fan Y, Mu JC, Gibeling G, Barr S, Asadi NB, Gerstein MB, et al. An ensemble approach to accurately detect somatic mutations using SomaticSeq. Genome Biol. 2015,16:197.

7. Chalmers ZR, Connelly CF, Fabrizio D, Gay L, Ali SM, Ennis R, Schrock A, Campbell B, Shlien A, Chmielecki J, et al. Analysis of 100,000 human cancer genomes reveals the landscape of tumor mutational burden. Genome Med. 2017,9:34.

8. Helleman J, Jansen MP, Span PN, van Staveren IL, Massuger LF, Meijer-van Gelder ME, Sweep FC, Ewing PC, van der Burg ME, Stoter G, et al. Molecular profiling of platinum resistant ovarian cancer. Int J Cancer. 2006,118:1963-71.

9. Niu B, Ye K, Zhang Q, Lu C, Xie M, McLellan MD, Wendl MC, Ding L. MSIsensor: microsatellite instability detection using paired tumor-normal sequence data. Bioinformatics. 2014,30:1015-6.

10. Marquard AM, Eklund AC, Joshi T, Krzystanek M, Favero F, Wang ZC, Richardson AL, Silver DP, Szallasi Z, Birkbak NJ. Pan-cancer analysis of genomic scar signatures associated with homologous recombination deficiency suggests novel indications for existing cancer drugs. Biomark Res. 2015,3:9.

11. Telli ML, Timms KM, Reid J, Hennessy B, Mills GB, Jensen KC, Szallasi Z, Barry WT, Winer EP, Tung NM, et al. Homologous Recombination Deficiency (HRD) Score Predicts Response to Platinum-Containing Neoadjuvant Chemotherapy in Patients with Triple-Negative Breast Cancer. Clin Cancer Res. 2016,22:3764-73.

12. Chakravarty D, Gao J, Phillips SM, Kundra R, Zhang H, Wang J, Rudolph JE, Yaeger R, Soumerai T, Nissan MH, et al. OncoKB: A Precision Oncology Knowledge Base. JCO Precis Oncol. 2017,2017.

13. Andre F, Bachelot T, Commo F, Campone M, Arnedos M, Dieras V, Lacroix-Triki M, Lacroix L, Cohen P, Gentien D, et al. Comparative genomic hybridisation array and DNA sequencing to direct treatment of metastatic breast cancer: a multicentre, prospective trial (SAFIR01/UNICANCER). Lancet Oncol. 2014,15:267-74.

14. Le Tourneau C, Delord JP, Goncalves A, Gavoille C, Dubot C, Isambert N, Campone M, Tredan O, Massiani MA, Mauborgne C, et al. Molecularly targeted therapy based on tumour molecular profiling versus conventional therapy for advanced cancer (SHIVA): a multicentre, open-label, proof-of-concept, randomised, controlled phase 2 trial. Lancet Oncol. 2015,16:1324-34.

15. Von Hoff DD, Stephenson JJ, Jr., Rosen P, Loesch DM, Borad MJ, Anthony S, Jameson G, Brown S, Cantafio N, Richards DA, et al. Pilot study using molecular profiling of patients' tumors to find potential targets and select treatments for their refractory cancers. J Clin Oncol. 2010,28:4877-83.

16. Mateo J, Chakravarty D, Dienstmann R, Jezdic S, Gonzalez-Perez A, Lopez-Bigas N, Ng CKY, Bedard PL, Tortora G, Douillard JY, et al. A framework to rank genomic alterations as targets for cancer precision medicine: the ESMO Scale for Clinical Actionability of molecular Targets (ESCAT). Ann Oncol. 2018,29:1895-902.

17. Benjamini Y, Hochberg Y. Controlling the false discovery rate: a practical and powerful approach to multiple tetsing. J R Statist Soc B. 1995,57:289-300.


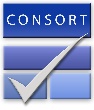
CONSORT 2010 checklist of information to include when reporting a randomised trial*

| Section/Topic | Item No | Checklist item | Reported on page No of the revised version |
| --- | --- | --- | --- |
| Title and abstract | | | |
|  | 1a | Identification as a trial in the title | 1 |
|  | 1b | Structured summary of trial design, methods, results, and conclusions (for specific guidance see CONSORT for abstracts) | 3 |
| Introduction | | | |
| Background and objectives | 2a | Scientific background and explanation of rationale | 5 |
|  | 2b | Specific objectives or hypotheses | 6 |
| Methods | | | |
| Trial design | 3a | Description of trial design (such as parallel, factorial) including allocation ratio | 7 |
|  | 3b | Important changes to methods after trial commencement (such as eligibility criteria), with reasons | 7 |
| Participants | 4a | Eligibility criteria for participants | 7 |
|  | 4b | Settings and locations where the data were collected | 7 |
| Interventions | 5 | The interventions for each group with sufficient details to allow replication, including how and when they were actually administered | 7 |
| Outcomes | 6a | Completely defined pre-specified primary and secondary outcome measures, including how and when they were assessed | 10 |
|  | 6b | Any changes to trial outcomes after the trial commenced, with reasons | 10 |
| Sample size | 7a | How sample size was determined | 10 |
|  | 7b | When applicable, explanation of any interim analyses and stopping guidelines | 10 |
| Randomisation: |  |  | Not randomized |
| Sequence generation | 8a | Method used to generate the random allocation sequence | Not randomized |
|  | 8b | Type of randomisation; details of any restriction (such as blocking and block size) | Not randomized |
| Allocation concealment mechanism | 9 | Mechanism used to implement the random allocation sequence (such as sequentially numbered containers), describing any steps taken to conceal the sequence until interventions were assigned | Not randomized |
| Implementation | 10 | Who generated the random allocation sequence, who enrolled participants, and who assigned participants to interventions | Not randomized |
| Blinding | 11a | If done, who was blinded after assignment to interventions (for example, participants, care providers, those assessing outcomes) and how | Not randomized |
|  | 11b | If relevant, description of the similarity of interventions | Not randomized |
| Statistical methods | 12a | Statistical methods used to compare groups for primary and secondary outcomes | 10 |
|  | 12b | Methods for additional analyses, such as subgroup analyses and adjusted analyses | 10 |
| Results | | | |
| Participant flow (a diagram is strongly recommended) | 13a | For each group, the numbers of participants who were randomly assigned, received intended treatment, and were analysed for the primary outcome | 11 and Fig. 1 |
|  | 13b | For each group, losses and exclusions after randomisation, together with reasons | 11 |
| Recruitment | 14a | Dates defining the periods of recruitment and follow-up | 11 |
|  | 14b | Why the trial ended or was stopped | 11 and Add. File 1 |
| Baseline data | 15 | A table showing baseline demographic and clinical characteristics for each group | Table 1 |
| Numbers analysed | 16 | For each group, number of participants (denominator) included in each analysis and whether the analysis was by original assigned groups | Not randomized |
| Outcomes and estimation | 17a | For each primary and secondary outcome, results for each group, and the estimated effect size and its precision (such as 95% confidence interval) | Not randomized |
|  | 17b | For binary outcomes, presentation of both absolute and relative effect sizes is recommended | Not randomized |
| Ancillary analyses | 18 | Results of any other analyses performed, including subgroup analyses and adjusted analyses, distinguishing pre-specified from exploratory | 13-17 |
| Harms | 19 | All important harms or unintended effects in each group (for specific guidance see CONSORT for harms) | 11 |
| Discussion | | | |
| Limitations | 20 | Trial limitations, addressing sources of potential bias, imprecision, and, if relevant, multiplicity of analyses | 26 |
| Generalisability | 21 | Generalisability (external validity, applicability) of the trial findings | 19-25 |
| Interpretation | 22 | Interpretation consistent with results, balancing benefits and harms, and considering other relevant evidence | 19-25 |
| Other information | | |  |
| Registration | 23 | Registration number and name of trial registry | NCT02342158 (ClinicalTrials.gov) |
| Protocol | 24 | Where the full trial protocol can be accessed, if available | Journal website |
| Funding | 25 | Sources of funding and other support (such as supply of drugs), role of funders | label Ligue EL2016 (DB) and EL2019 (FB), Ruban Rose (DB), Association La Marie-Do (FB), Fondation Groupe EDF (DB), and Centre d’Investigations Cliniques (CICp1409) |

*We strongly recommend reading this statement in conjunction with the CONSORT 2010 Explanation and Elaboration for important clarifications on all the items. If relevant, we also recommend reading CONSORT extensions for cluster randomised trials, non-inferiority and equivalence trials, non-pharmacological treatments, herbal interventions, and pragmatic trials. Additional extensions are forthcoming: for those and for up to date references relevant to this checklist, see [www.consort-statement.org](http://www.consort-statement.org).
