# Supplementary material for: Prospective high-throughput genome profiling of advanced cancers: results of the PERMED-01 clinical trial
Source: Genome Med. 2021 May 18;13:87. doi: 10.1186/s13073-021-00897-9 (PMC8132379; doi:10.1186/s13073-021-00897-9)
Supplement: Supplementary file 4 — Additional file 4: Supplementary Figures. Contains four Supplementary Figures showing the cancer types profiled and further NGA and aCGH results. [file 13073_2021_897_MOESM4_ESM.pptx]

## Slide 1
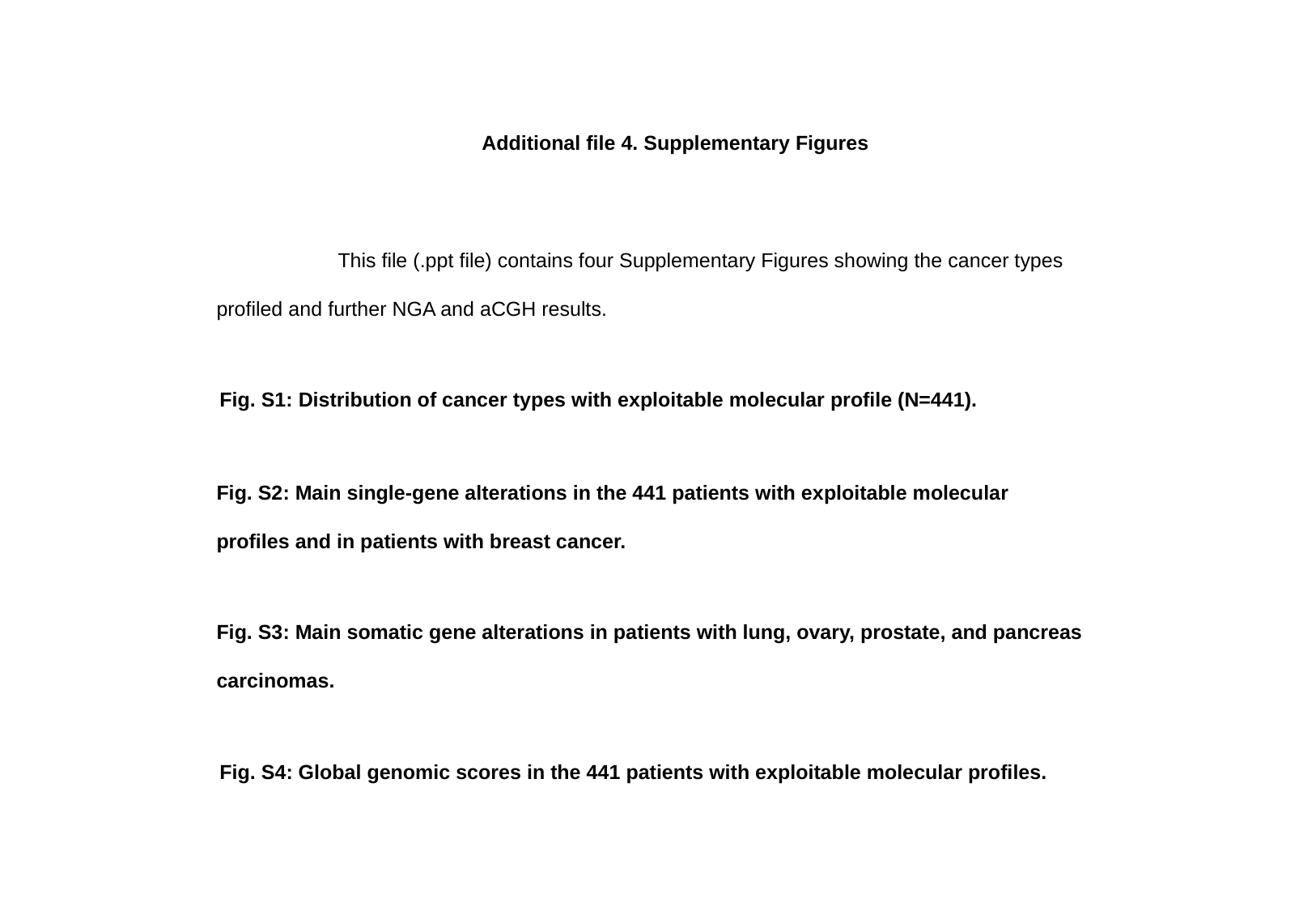

Additional file 4. Supplementary Figures
	This file (.ppt file) contains four Supplementary Figures showing the cancer types profiled and further NGA and aCGH results.
Fig. S1: Distribution of cancer types with exploitable molecular profile (N=441).
Fig. S2: Main single-gene alterations in the 441 patients with exploitable molecular profiles and in patients with breast cancer.
Fig. S3: Main somatic gene alterations in patients with lung, ovary, prostate, and pancreas carcinomas.
Fig. S4: Global genomic scores in the 441 patients with exploitable molecular profiles.

## Slide 2
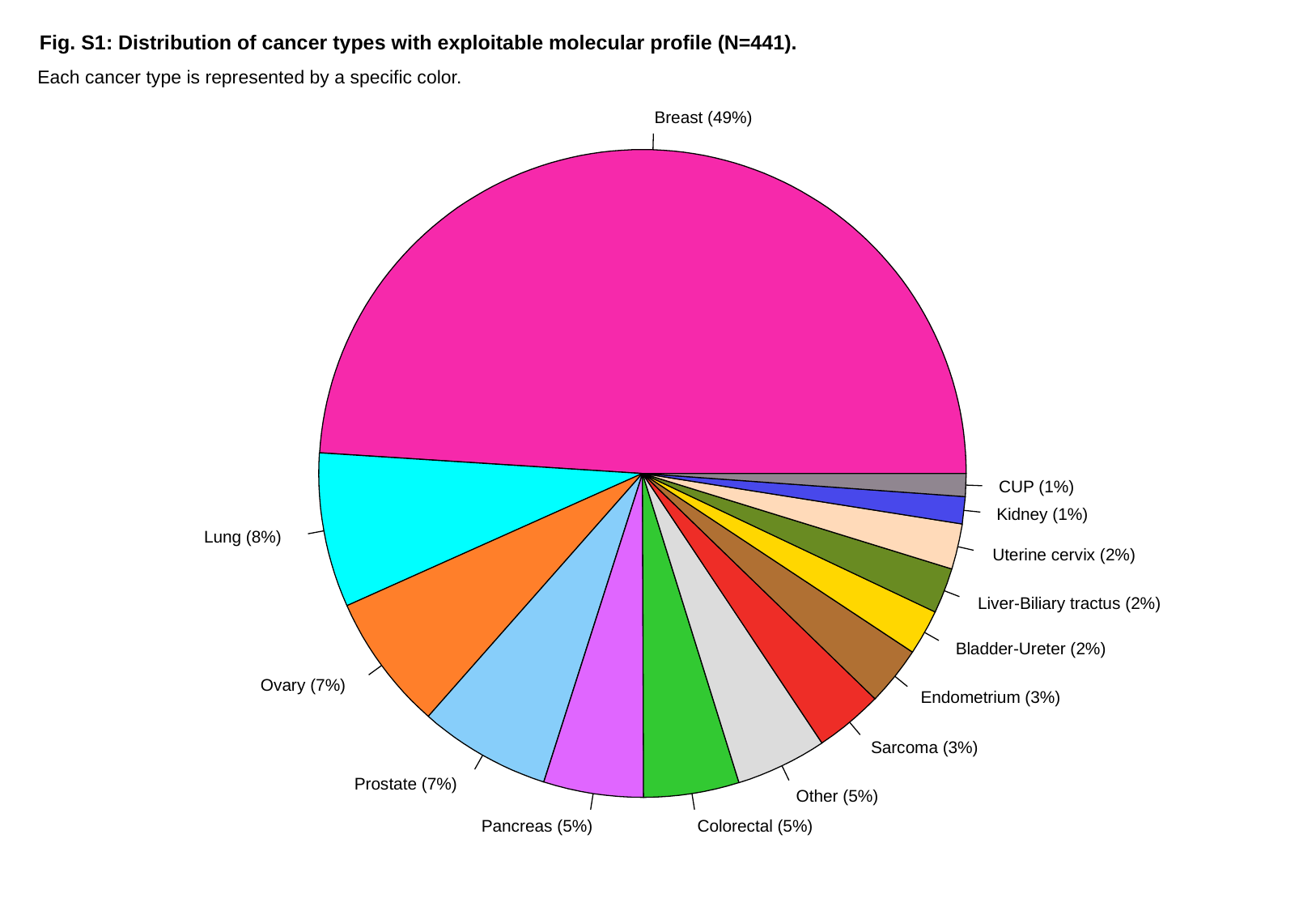

Fig. S1: Distribution of cancer types with exploitable molecular profile (N=441).
Each cancer type is represented by a specific color.
Breast (49%)
CUP (1%)
Kidney (1%)
Lung (8%)
Uterine cervix (2%)
Liver-Biliary tractus (2%)
Bladder-Ureter (2%)
Ovary (7%)
Endometrium (3%)
Sarcoma (3%)
Prostate (7%)
Other (5%)
Pancreas (5%)
Colorectal (5%)

## Slide 3
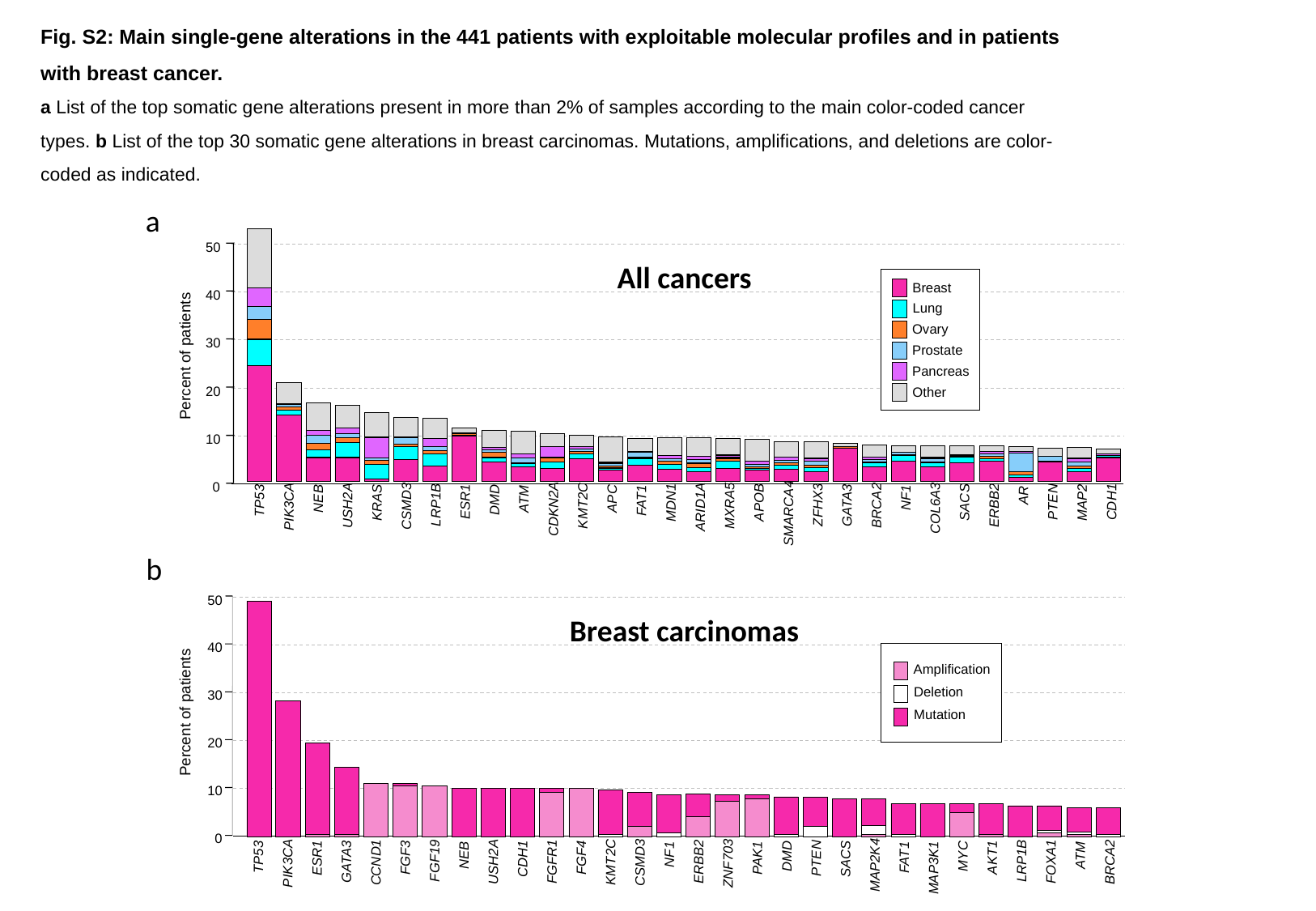

Fig. S2: Main single-gene alterations in the 441 patients with exploitable molecular profiles and in patients with breast cancer.
a List of the top somatic gene alterations present in more than 2% of samples according to the main color-coded cancer types. b List of the top 30 somatic gene alterations in breast carcinomas. Mutations, amplifications, and deletions are color-coded as indicated.
a
AR
NF1
NEB
APC
ATM
DMD
TP53
FAT1
ESR1
PTEN
CDH1
KRAS
SACS
MAP2
APOB
MDN1
ZFHX3
LRP1B
GATA3
ERBB2
USH2A
BRCA2
KMT2C
MXRA5
CSMD3
PIK3CA
ARID1A
COL6A3
CDKN2A
SMARCA4
50
40
30
20
10
0
Percent of patients
All cancers
Breast
Lung
Ovary
Prostate
Pancreas
Other
b
50
40
30
20
10
0
Percent of patients
NF1
NEB
ATM
MYC
DMD
TP53
FAT1
AKT1
FGF3
FGF4
PAK1
ESR1
PTEN
CDH1
SACS
FGF19
LRP1B
FGFR1
GATA3
ERBB2
FOXA1
USH2A
BRCA2
KMT2C
CCND1
CSMD3
PIK3CA
ZNF703
MAP2K4
MAP3K1
Breast carcinomas
Amplification
Deletion
Mutation

## Slide 4
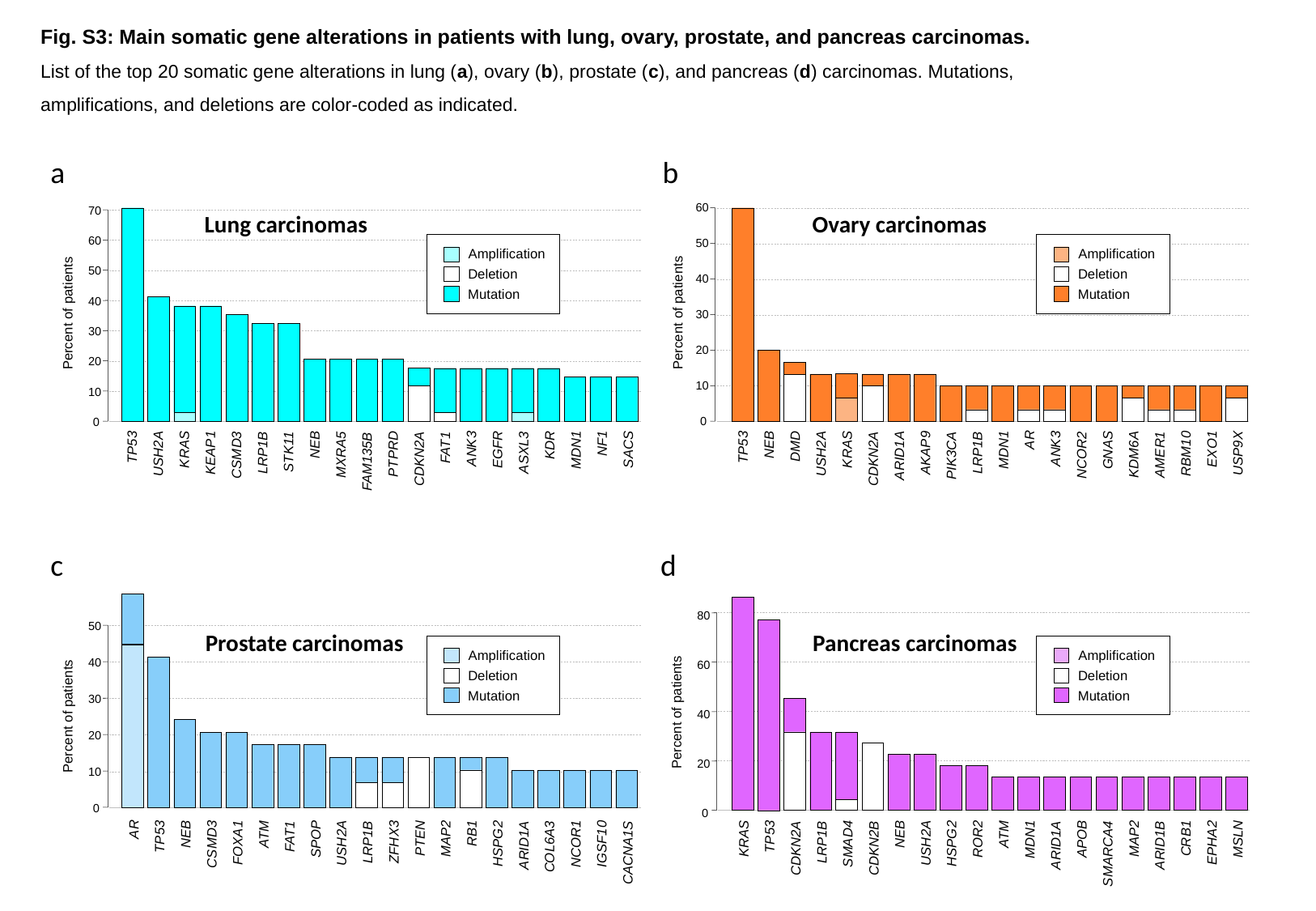

Fig. S3: Main somatic gene alterations in patients with lung, ovary, prostate, and pancreas carcinomas.
List of the top 20 somatic gene alterations in lung (a), ovary (b), prostate (c), and pancreas (d) carcinomas. Mutations, amplifications, and deletions are color-coded as indicated.
a
b
60
50
40
30
20
10
0
AR
NEB
DMD
TP53
ANK3
EXO1
KRAS
MDN1
GNAS
LRP1B
AKAP9
USP9X
RBM10
USH2A
KDM6A
AMER1
NCOR2
PIK3CA
ARID1A
CDKN2A
Percent of patients
Amplification
Deletion
Mutation
Lung carcinomas
Ovary carcinomas
70
60
50
40
Percent of patients
30
20
10
0
NF1
NEB
KDR
TP53
FAT1
ANK3
KRAS
SACS
EGFR
MDN1
STK11
ASXL3
LRP1B
KEAP1
USH2A
PTPRD
MXRA5
CSMD3
CDKN2A
FAM135B
Amplification
Deletion
Mutation
c
d
50
40
30
Percent of patients
20
10
0
AR
RB1
NEB
ATM
TP53
FAT1
PTEN
MAP2
SPOP
ZFHX3
LRP1B
FOXA1
USH2A
HSPG2
IGSF10
CSMD3
NCOR1
ARID1A
COL6A3
CACNA1S
Amplification
Deletion
Mutation
NEB
ATM
TP53
CRB1
KRAS
MAP2
MSLN
ROR2
APOB
MDN1
LRP1B
EPHA2
USH2A
HSPG2
SMAD4
ARID1A
ARID1B
CDKN2A
CDKN2B
SMARCA4
80
60
Percent of patients
40
20
0
Amplification
Deletion
Mutation
Prostate carcinomas
Pancreas carcinomas

## Slide 5
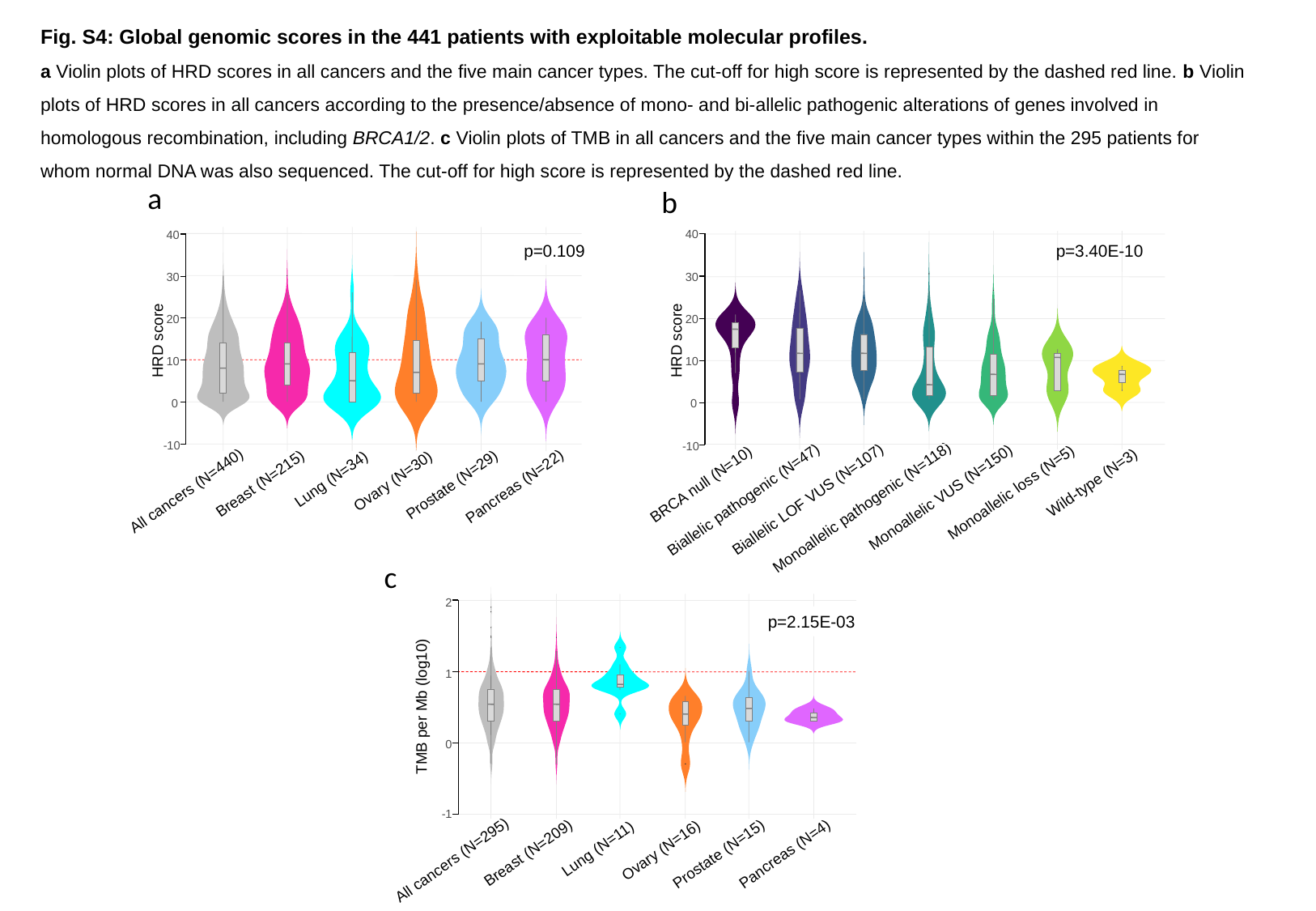

Fig. S4: Global genomic scores in the 441 patients with exploitable molecular profiles.
a Violin plots of HRD scores in all cancers and the five main cancer types. The cut-off for high score is represented by the dashed red line. b Violin plots of HRD scores in all cancers according to the presence/absence of mono- and bi-allelic pathogenic alterations of genes involved in homologous recombination, including BRCA1/2. c Violin plots of TMB in all cancers and the five main cancer types within the 295 patients for whom normal DNA was also sequenced. The cut-off for high score is represented by the dashed red line.
a
40
p=0.109
30
20
HRD score
10
0
-10
Lung (N=34)
Ovary (N=30)
Breast (N=215)
Prostate (N=29)
Pancreas (N=22)
All cancers (N=440)
b
40
p=3.40E-10
30
20
HRD score
10
0
-10
Wild-type (N=3)
BRCA null (N=10)
Monoallelic loss (N=5)
Monoallelic VUS (N=150)
Biallelic LOF VUS (N=107)
Biallelic pathogenic (N=47)
Monoallelic pathogenic (N=118)
c
p=2.15E-03
Lung (N=11)
Ovary (N=16)
Breast (N=209)
Pancreas (N=4)
Prostate (N=15)
All cancers (N=295)
2
1
TMB per Mb (log10)
0
-1
